# Statin-dependent and -independent pathways are associated with major adverse cardiovascular events in people with HIV

**DOI:** 10.1172/JCI196021

**Published:** 2025-09-09

**Authors:** Márton Kolossváry, Irini Sereti, Markella V. Zanni, Carl J. Fichtenbaum, Judith A. Aberg, Gerald S. Bloomfield, Carlos D. Malvestutto, Judith S. Currier, Sarah M. Chu, Marissa R. Diggs, Alex B. Lu, Christopher deFilippi, Borek Foldyna, Sara McCallum, Craig A. Sponseller, Michael T. Lu, Pamela S. Douglas, Heather J. Ribaudo, Steven K. Grinspoon

**Affiliations:** 1 Metabolism Unit, Massachusetts General Hospital and Harvard Medical School, Boston, Massachusetts, USA.; 2 Laboratory of Immunoregulation, National Institute of Allergy and Infectious Diseases (NIAID), NIH, Bethesda, Maryland, USA.; 3 Division of Infectious Diseases, University of Cincinnati College of Medicine, Cincinnati, Ohio, USA.; 4 Division of Infectious Diseases, Icahn School of Medicine at Mount Sinai, New York, New York, USA.; 5 Department of Medicine, Duke Global Health Institute and Duke Clinical Research Institute, Duke University School of Medicine, Durham, North Carolina, USA.; 6 Division of Infectious Diseases, Ohio State University Medical Center, Columbus, Ohio, USA.; 7 Division of Infectious Diseases, David Geffen School of Medicine, UCLA, Los Angeles, California, USA.; 8 Inova Schar Heart and Vascular, Falls Church, Virginia, USA.; 9 Cardiovascular Imaging Research Center, Massachusetts General Hospital and Harvard Medical School, Boston, Massachusetts, USA.; 10 Kowa Pharmaceuticals America Inc., Montgomery, Alabama, USA.; 11 Duke Clinical Research Institute, Duke University School of Medicine, Durham, North Carolina, USA.; 12 Center for Biostatistics in AIDS Research, Harvard T.H. Chan School of Public Health, Boston, Massachusetts, USA.

**Keywords:** AIDS/HIV, Cardiology, Inflammation, Cardiovascular disease, Machine learning, Proteomics

## Abstract

**BACKGROUND:**

Statin therapy lowers the risk of major adverse cardiovascular events (MACE) among people with HIV (PWH). Residual risk pathways contributing to excess MACE beyond LDL-cholesterol (LDL-C) are not well understood. Our objective was to evaluate the association of statin-responsive and other inflammatory and metabolic pathways with MACE in the Randomized Trial to Prevent Vascular Events in HIV (REPRIEVE).

**METHODS:**

Cox proportional hazards models were used to assess the relationship between MACE and proteomics measurements at study entry and year 2, adjusting for time-updated statin use and the baseline 10-year atherosclerotic cardiovascular disease risk score. We built a machine-learning (ML) model to predict MACE using baseline protein values with significant associations.

**RESULTS:**

For 765 individuals (age: 50.8 ± 5.9 years, 82% men, 18% women), among 7 proteins changing with statin versus placebo, angiopoietin-related protein 3 (ANGPTL3) related most strongly to MACE (adjusted HR [aHR]: 2.31 per 2-fold-higher levels; 95% CI: 1.11–4.80; *P* = 0.03), such that lower levels of ANGPTL3 achieved with statin therapy were associated with lower MACE risk. Among 248 proteins that did not change in response to statin therapy, 26 were associated with MACE at a FDR below 0.05. These proteins represented a predominantly humoral immune response, leukocyte chemotaxis, and cytokine pathways. Our proteomics ML model achieved a 10-fold cross-validated concordance index (C-index) of 0.74 ± 0.11 to predict MACE, improving on models using traditional risk prediction scores only (C-index: 0.61 ± 0.18).

**CONCLUSIONS:**

ANGPTL3, as well as key inflammatory pathways, may contribute to a residual risk of MACE among PWH, beyond LDL-C.

**TRIAL REGISTRATION:**

ClinicalTrials.gov: NCT02344290.

**FUNDING:**

NIH, Kowa Pharmaceuticals America, Gilead Sciences, ViiV Healthcare.

## Abstract 2

People with HIV may face additional heart disease event risk from a protein called ANGPTL3, which affects LDL processing beyond typical lipid problems.

## Introduction

People with HIV (PWH) are known to be at higher risk of major adverse cardiac events (MACE) (1–3). The Randomized Trial to Prevent Vascular Events in HIV (REPRIEVE) demonstrated a 36% reduction in MACE rates among PWH who were randomized to pitavastatin treatment as compared with placebo over a median of 5.6 years (4, 5). The effects on MACE appeared larger than could be accounted for with changes in low-density lipoprotein cholesterol (LDL-C) alone (4). To assess statin effects beyond LDL-C, we previously assessed 255 proteins on 3 Olink platforms (Cardiovascular III, Immuno-oncology, and Cardiometabolic panels) within the REPRIEVE mechanistic substudy, which showed that specific proteins representing inflammatory and other lipid pathways beyond LDL-C changed in response to statin therapy. Notably, among these proteins, the levels of angiopoietin-related protein 3 (ANGPTL3), a protein known to be associated with cardiovascular disease (CVD) in the general population, decreased with statin therapy (6, 7).

Our objective in this analysis was to further leverage the REPRIEVE mechanistic study to assess the relationship of relevant protein pathways to MACE in PWH. We first evaluated the relationship of statin responsive pathways to incident MACE. Second, we sought to identify inflammatory and immunological markers of residual cardiovascular risk that are associated with MACE in unadjusted and adjusted models accounting for atherosclerotic cardiovascular disease (ASCVD) risk and statin use. Together, these data inform the field of relevant mechanistic pathways and potential therapeutic targets beyond the lowering of LDL-C to prevent MACE in PWH.

## Results

### Study population.

**Figure 1:**
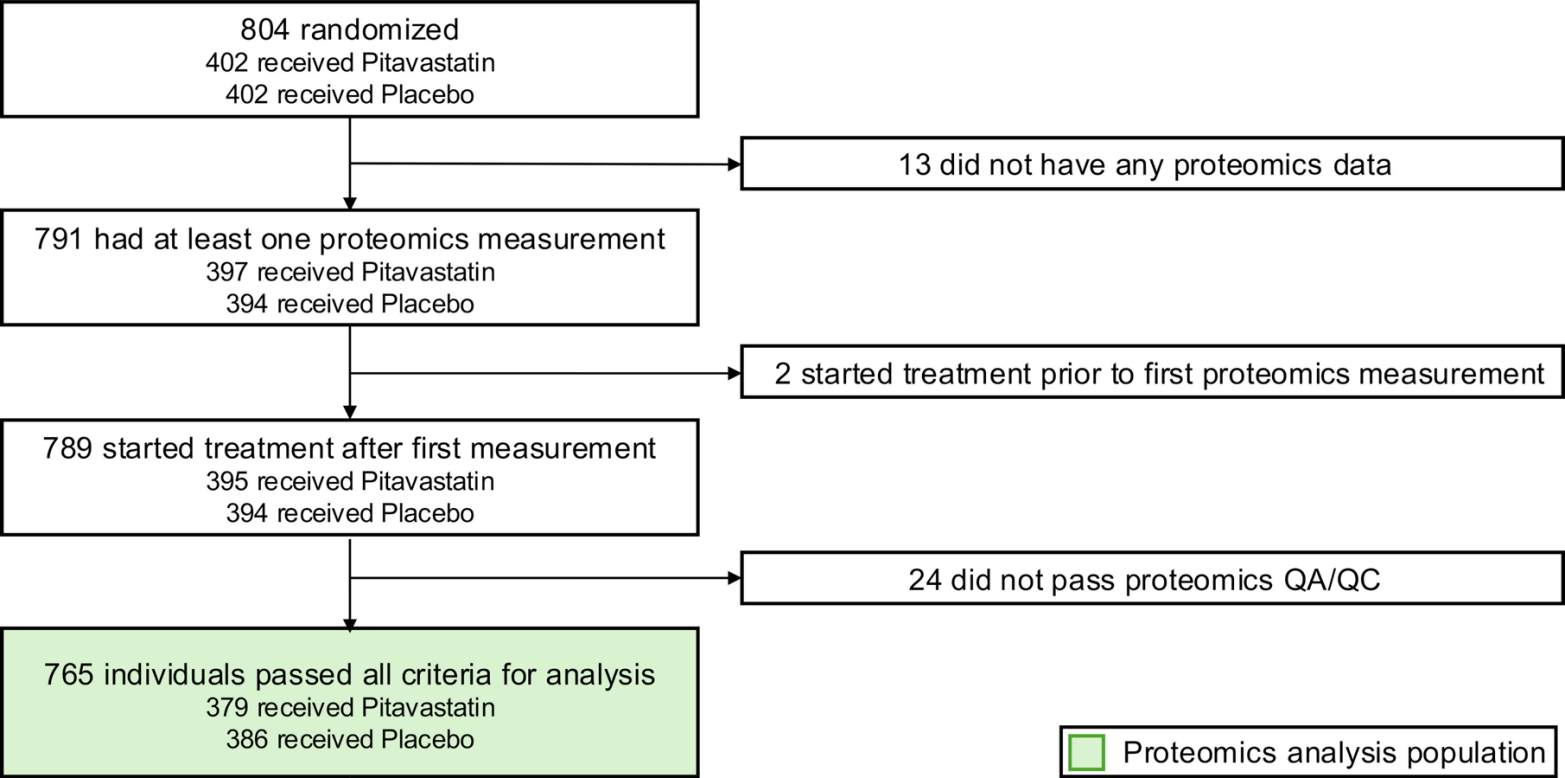
CONSORT diagram of individuals included in the current analysis. Overall, 765 individuals were included in our analyses to evaluate the association between proteomics markers and MACE. QA, quality assurance; QC, quality control.

Between 2015 and 2018, a total of 804 participants were randomized in the REPRIEVE mechanistic substudy. For our current analysis, 39 individuals were excluded because of a lack of availability, sampling time, and/or quality of proteomics measurements, resulting in 765 individuals (379 randomized to pitavastatin and 386 to placebo, respectively) being included in our analyses (Figure 1). The average age of our participants was 50.8 ± 5.9 years, and 82% (*n* = 631) were men and 18% (*n* = 134) were women. The individuals were predominantly White, non-Hispanic participants or Latino participants. The mean duration of antiretroviral therapy (ART) was 11.8 ± 6.6 years at baseline. The average 10-year ASCVD risk score for the individuals was 5.0% ± 3.1%, and the baseline levels of LDL-C were 108.2 ± 29.8 mg/dL and 133.7 ± 83.2 mg/dL for triglycerides (Table 1).

Over the average follow-up of 5.8 ± 1.9 years in the mechanistic substudy (median: 6.2 years, IQR: 5.5; 7.1 years), 33 participants experienced a MACE. Participants who experienced MACE were older (53.2 ± 5.4 vs. 50.7 ± 6.0 years, respectively), had higher 10-year ASCVD risk scores (6.9 ± 3.6 vs. 5.0 ± 3.1, respectively), were more likely to be a current smoker (52% vs. 24%, respectively), and had higher high-sensitivity C-reactive protein levels (4.5 ± 3.3 vs. 2.8 ± 2.9 mg/L, respectively, Table 1). In our analysis population, 20 individuals had a hard MACE (*n* = 6 cardiovascular deaths, *n* = 5 myocardial infarctions, *n* = 9 strokes, Supplemental Table 1; supplemental material available online with this article; https://doi.org/10.1172/JCI196021DS1).

### Association of statin-responsive proteins with MACE.

Among a previously identified set of 7 proteins changing in response to pitavastatin treatment (6), 4 proteins showed decreased expression in response to statin therapy, and 3 showed increased expression. In our time-updated Cox models utilizing both baseline and 2-year proteomics measurements, only ANGPTL3 had a significant association with MACE (HR: 2.20 per 2-fold higher protein level; 95% CI: 1.04–4.67; *P* = 0.04, which corresponds to a HR of 1.40 and a 95% CI of 1.02–1.92 per population SD increase in normalized protein expression [NPX] values), such that lower levels of ANGPTL3 achieved with statin therapy were associated with lower MACE rates. Adjusting our models for 10-year ASCVD risk score and statin use resulted in similar results (HR: 2.31; 95% CI: 1.11–4.80; *P* = 0.03, which corresponds to a HR of 1.43 and a 95% CI of 1.04–1.96 per population SD increase in NPX values). No other proteins were significantly associated with MACE (Table 2). ANGPTL3 was also significantly associated with hard MACE (HR: 3.27; 95% CI: 1.32-8.13; *P* = 0.01, which corresponds to a HR of 1.66 and a 95% CI of 1.13–2.45 per population SD increase in NPX values), and this relationship remained consistent in adjusted models accounting for a 10-year ASCVD risk score and statin use (HR: 3.46; 95% CI: 1.43–8.53; *P* = 0.005, which corresponds to a HR of 1.70 and a 95% CI of 1.17–1.48 per population SD increase in NPX values). Supplemental analyses using statin randomization rather than time-updated statin use showed similar results (Supplemental Table 2).

In our study population, individuals randomized to statin therapy demonstrated an 11.1% (95% CI: –15.3%; –6.8%, *P* < 0.0001) reduction in ANGPTL3 levels, 15.3 mg/dL (95% CI: –27.4 mg/dL; –3.1 mg/dL; *P* = 0.013) reduction in triglyceride levels and a 27.8 mg/dL (95%CI: –32.5 mg/dL; –23.1 mg/dL; *P* < 0.001) reduction in LDL-C levels from baseline to 2-year follow-up compared with those randomized to placebo. In mediation analysis, 29.8% (95% CI: 11.6%; 52.5%) of the association between statin therapy and triglyceride levels and 5.9% (95% CI: 1.87%; 10.8%) of the association between statin therapy and LDL-C levels appeared mediated through the effect of statins on ANGPTL3. We thus further adjusted our analyses relating ANGPTL3 to MACE for time-updated LDL-C and triglyceride levels, which revealed similar results and a persistent relationship of ANGPTL3 to MACE (Supplemental Table 3).

### Inflammatory, immune-oncological, and cardiometabolic proteomics markers related to MACE in residual risk analyses.

**Figure 2:**
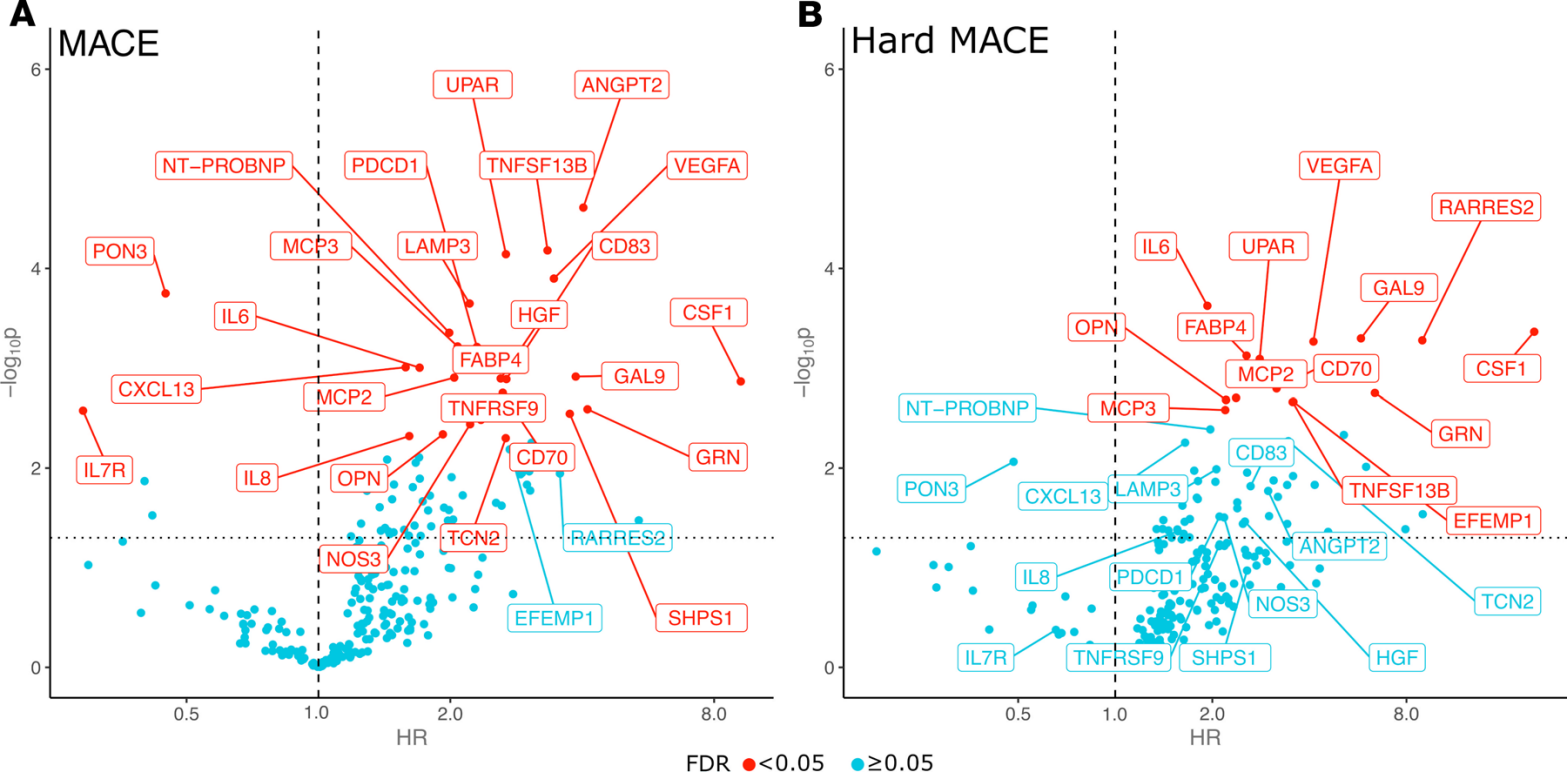
Association between proteomics markers and MACE or hard MACE. (**A**) Volcano plot of HRs and *P* values for given proteins and MACE. HRs were estimated from Cox proportional hazards models using both potential proteomics measurements including statin use as a time-dependent covariate and 10-year baseline ASCVD risk score. HRs are per protein doubling. Red proteins indicate associations at an FDR below 0.05 level. The *x* axis is on a log_2_ scale. Dotted line indicates a nominal *P* value of 0.05. Dashed line indicates a HR of 1. (**B**) Volcano plot of HRs and *P* values for given proteins and hard MACE. Hard MACE is defined as cardiovascular death, myocardial infarction, or stroke. HRs were estimated from Cox proportional hazards models using both potential proteomics measurements including statin use as a time-dependent covariate and baseline 10-year ASCVD risk score. HRs are per protein doubling. Red proteins indicate associations at an FDR below 0.05. The *x* axis is on a log_2_ scale. Dotted line indicates a nominal *P* value of 0.05. Dashed line indicates a HR of 1. ANGPT2, angiopoietin-2; CD70, CD70 antigen; CD83, CD83 antigen; CSF1, processed macrophage colony-stimulating factor 1; CXCL13, C-X-C motif chemokine 13; FABP4, fatty acid–binding protein, adipocyte; GAL9, galectin-9; GRN, paragranulin; HGF, hepatocyte growth factor α chain; MCP2, C-C motif chemokine 8; MCP3, C-C motif chemokine 7; NOS3, nitric oxide synthase, endothelial; NT-PROBNP, NT-proBNP; OPN, osteopontin; PDCD1, programmed cell death protein 1; SHPS1, tyrosine-protein phosphatase non-receptor-type substrate 1; TCN2, transcobalamin-2; TNFRSF9, TNF receptor superfamily member 9; TNFSF13B, TNF ligand superfamily member 13b, membrane form; VEGFA, vascular endothelial growth factor A.

Among the 248 remaining proteins in the proteomics panels (Supplemental Figure 1), which did not significantly change with statin treatment, in our time-updated Cox models utilizing both baseline and 2-year proteomics measurements, 26 proteins were associated with MACE at an FDR of less than 0.05 after adjusting for statin use and 10-year ASCVD risk scores. Among these, the receptor for IL-7 (IL-7R) (HR: 0.29; 95% CI: 0.13; 0.65; *P* = 0.003, which corresponds to a HR of 0.59; 95% CI: 0.42–0.83 per population SD increase in NPX values) and paraoxonase 3 (PON3) (HR: 0.45; 95% CI: 0.29; 0.68; *P* = 0.0001, which corresponds to a HR of 0.55; 95% CI: 0.41–0.75 per population SD increase in NPX values) were associated with a lower risk of MACE. All other proteins were associated with an increased the risk of MACE. The effect sizes of the remaining 24 proteins ranged from a HR of 1.58–9.20 per protein doubling (Figure 2 and Supplemental Table 4). We observed similar results in supplemental analyses adjusting for statin randomization rather than time-updated statin use (Supplemental Figure 2). Numerically, we also observed similar results for hard MACE (Supplemental Table 5). Among the 12 proteins with an FDR of less than 0.05 association with hard MACE, 10 were also identified for MACE. Only EGF-containing, fibulin-like extracellular matrix protein 1 (EFEMP1) and retinoic acid receptor responder protein 2 (RARRES2) were identified as associated with hard MACE but did not reach statistical significance for the MACE outcome. Detailed results, protein names, and abbreviations for proteins identified in these analyses can be found in Table 3. In sensitivity analyses, further adjustment for LDL-C and triglyceride levels produced numerically similar results for all proteins (Supplemental Figure 3).

### Correlation among proteins related to MACE and relation to baseline clinical factors.

ANGPTL3 was related to multiple proteins and, most significantly, to the urokinase plasminogen activator surface receptor (UPAR) (ρ = 0.54). In contrast, other proteins related to MACE showed only a moderate correlation with each other. Proteins related to MACE were only weakly correlated with clinical factors and clinically assessed biomarkers. A detailed correlation heatmap can be found in Supplemental Figures 4 and 5.

### Protein-protein interaction analysis for non-statin-modifiable proteins.

**Figure 3:**
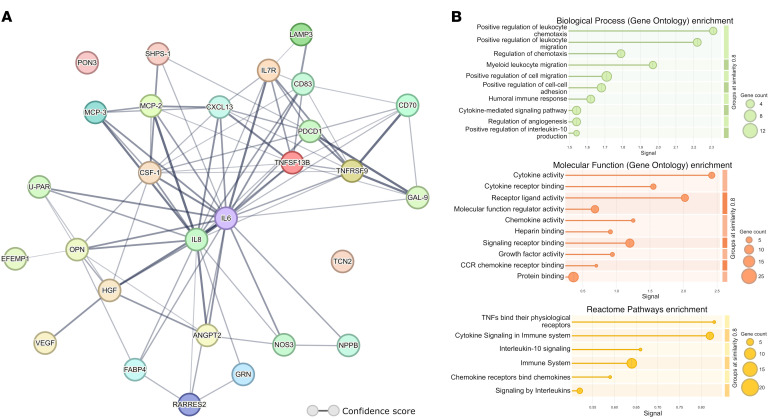
Protein-protein interaction and enrichment analysis among proteins showing a significant association with MACE or hard MACE. (**A**) Confidence-based protein interaction network using all available interactions sources. The width of the edges between the proteins represents the overall confidence score. The average number of connections between the protein nodes is 6.21, while IL-6 and IL-8 are connected to 21 and 18 proteins, respectively. (**B**) Gene Ontology and Reactome functional enrichments analysis. Gene Ontology biological processes, molecular function, and Reactome pathway enrichment all indicated increased representation of cytokine and chemokine processes and pathway functions within our significant proteins. CCR, chemokine-chemokine receptor.

For the 28 proteins showing a significant association with MACE or hard MACE at an FDR below 0.05 in residual risk analyses, we created an exploratory protein-protein interaction network to further understand the biological role of these proteins. All proteins except PON3 and transcobalamin-2 (TCN) were part of a single cluster (average number of connections: 6.21), where IL-6 and IL-8 were connected to the largest numbers of other proteins (*n* = 21 and *n* = 18, respectively, Figure 3). Exploratory Gene Ontology biological processes enrichment analysis showed an increased representation of leukocyte-associated chemotaxis and migration and humoral immune response. Gene Ontology molecular function enrichment analysis indicated increased cytokine- and chemokine-associated functions in our network. Reactome pathway analysis indicated increased enrichment of TNF, cytokine, and chemokine pathway functions within our significant proteins. Detailed enrichment results can be found in Figure 3, with the lists of proteins corresponding to each term in Supplemental Table 6.

### Diagnostic power of proteomics markers to predict MACE beyond traditional risk indices.

**Figure 4:**
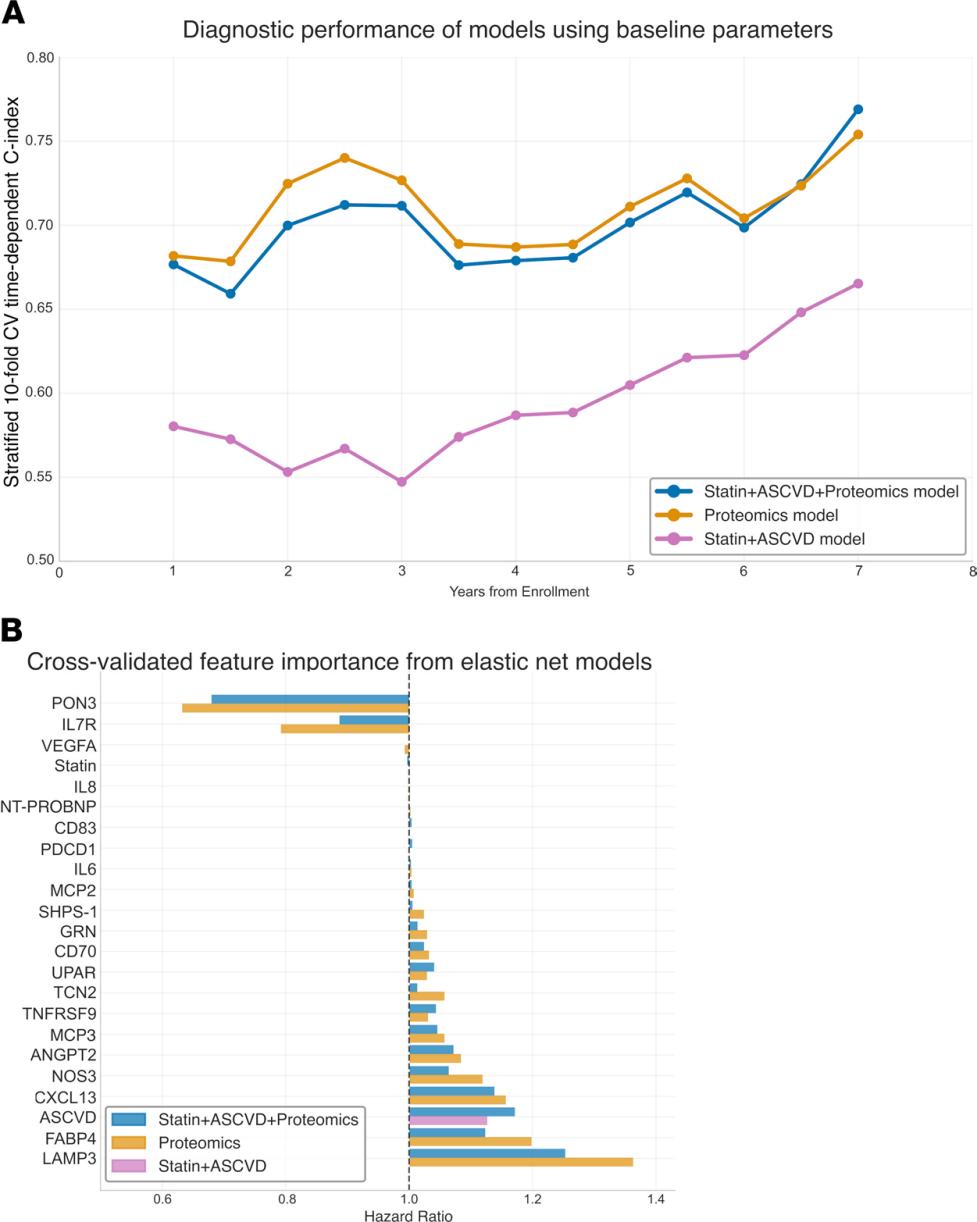
Cross-validated performance of significant proteins, unchanged with statin therapy, to predict MACE. (**A**) Stratified 10-fold cross-validated, time-dependent C-index of Cox elastic net machine learning models. Model 1: statin randomization + 10-year ASCVD risk score; model 2: baseline values of proteins associated with MACE; model 3: model 1 + model 2. (**B**) Importance of the features with a HR different from at least 1 in one of the models. Bars represent standardized average standardized HRs from the 10-fold cross-validation. CV, cross-validation.

To account for collinearity between the proteomics features, avoid overfitting, and evaluate the additive value of significant proteomics features, we built a machine learning elastic net Cox proportional hazards model using the significant proteomics markers, treatment randomization, and 10-year ASCVD risk scores, applying stratified 10-fold cross-validation. We developed 3 models. Model 1, incorporating statin randomization and the 10-year ASCVD risk score, achieved a 10-fold cross-validated concordance index (C-index) of 0.61 (SD = 0.18) for MACE. In contrast, model 2, which incorporated only baseline protein values, demonstrated a cross-validated C-index of 0.74 (SD = 0.12). Model 3, including ASCVD risk, statin randomization, and protein values, similarly demonstrated a cross-validated C-index of 0.74 (SD = 0.11). Lysosome-associated membrane glycoprotein 3 (LAMP3) had the largest HR among proteins positively associated with MACE, and PON3 had the lowest HR among proteins negatively associated with MACE (Figure 4).

## Discussion

In this analysis of the REPRIEVE mechanistic substudy, we identified several protein pathways related to MACE in PWH. Among these, *ANGPTL3* was notable, as it inhibits the uptake of triglyceride-rich lipoprotein remnants and was previously found to decrease in response to statin therapy among PWH in the REPRIEVE study. We now demonstrate that lower levels of *ANGPTL3* achieved with statin therapy were associated with lower MACE rates. We further identified an additional 26 proteins associated with MACE risk in residual risk analyses accounting for statin use. Enrichment analysis identified an overrepresentation of cytokine and chemokine processes and pathways representing a residual inflammatory risk within this cohort of PWH. A cross-validated machine learning model using the baseline values of these proteins outperformed a model including 10-year ASCVD risk and statin randomization.

ANGPTL3 is an endothelial lipase inhibitor secreted from the liver that inhibits lipoprotein lipase, important for overall lipid metabolism, regulation, and coronary artery disease (8, 9). By inhibiting lipoprotein lipase, ANGPTL3 interferes with the breakdown and clearance of triglycerides from triglyceride-rich particles including VLDL and chylomicrons. Individuals with loss-of-function variants in ANGPTL3 have lower triglyceride, HDL-C, and LDL-C levels and reduced incidence of CVD (10–13). Prior studies have shown that plasma ANGPTL3 levels were 15% lower in statin-treated familial hypercholesterolemia patients compared with statin-naive patients (14). In the Atherosclerosis Risk in the Communities (ARIC) study, ANGPTL3 levels were significantly reduced in a propensity score–matched analysis of statin users versus nonusers (15). We now further support these statin effects in PWH, showing in our prior analyses from REPRIEVE that ANGPTL3 was reduced by 11% in individuals randomized to the pitavastatin group compared with placebo-treated individuals (6). Also, the effect of statin therapy on circulating ANGPTL3 levels is supported by evidence from interventional trials in humans showing a reduction in liver expression of ANGPTL3 in response to statin therapy (16). However, the association between ANGPTL3 and MACE has not, to our knowledge, been previously established.

In REPRIEVE, baseline levels of LDL-C and triglycerides were only modestly increased and often normal. Despite modest baseline LDL-C levels, statin therapy had a major effect, reducing MACE by 36% in REPRIEVE. In the present study, ANGPTL3 appeared to mediate, in part, statin-related changes in triglyceride levels and, to a lesser extent, LDL-C levels, consistent with the known effects of ANGPTL3 to primarily regulate triglyceride levels. Furthermore, the HRs of MACE and hard MACE were increased with higher ANGPTL3 levels, such that lower levels of ANGPTL3 achieved with statin therapy were associated with reduced MACE rates. Of note, these relationships remained consistent when controlling for the 10-year ASCVD risk score, and also in sensitivity analyses controlling for LDL-C and triglyceride levels, suggesting this may be a direct effect related to ANGPTL3 effects on the vasculature independent of effects on lipid levels.

Given that the effects of ANGPTL3 reduction on LDL-C have been shown in LDL receptor–deficient mice (13), strategies to improve ANGPTL3 may be additive to statin effects. In addition, multiple prior studies involving PWH have shown insulin resistance in addition to hypertriglyceridemia, both of which are also known to be regulated by ANGPTL3 (17, 18). Emerging new antisense oligonucleotides, monoclonal antibodies, and gene-editing solutions to reduce ANGPTL3 levels have shown promise to reduce triglycerides, LDL-C levels, and, importantly, coronary artery lesion size (8, 9, 19), suggesting a mechanism whereby direct modulation of ANGPTL3 might affect subsequent MACE. Analyses between people with and without HIV have not shown significant differences in circulating ANGPTL3 levels, suggesting that our results may not be specific to PWH (20). Future studies are now needed to validate our findings linking ANGPTL3 to MACE and to assess the potential beneficial effects of new therapeutics targeting ANGPTL3 on clinical outcomes in PWH and the general population.

This proteomics analysis informs our understanding of pathways potentially contributing to MACE beyond LDL-C in PWH. These data on almost 300 proteins chosen from key cardiometabolic, immune, and inflammatory domains highlight the notion that many critical pathways may be perturbed in ART-treated PWH, independent of ASCVD risk and statin use, contributing to ongoing low-grade inflammation and immune activation and MACE. Prior work has shown that HIV persists in viral reservoirs in T cells and macrophages, despite stable ART, contributing to this phenomenon (21). The current work helps to define the particular pattern of proteins and pathways related to MACE among PWH receiving effective ART.

In our exploratory enrichment analysis, biomarkers found to be related to MACE in this study represent key related pathways in cytokine regulation, immune response, and chemotaxis. For example, chemotactic factors such as CXCL13 and growth factors driving myelopoiesis such as CSF1 were tightly interconnected with a central positioning of IL-6 and IL-8, both cytokines signifying innate, specifically myeloid immune activation with a known prominent role in atherosclerosis in network analyses. It is of interest to note that in the Canakinumab Antiinflammatory Thrombosis Outcome Study (CANTOS), decreases in IL-6 were associated with the observed clinical benefit both in terms of MACE and cancer, and it is not surprising that IL-6 targeting is now being pursued in atherosclerosis (22). In PWH, the presence of IL-6 and also the UPAR has been linked to non-AIDS malignancies as well as chronic kidney disease or osteoporosis (23, 24). The strong relationship between ANGTPL3 and the UPAR is notable, given the central role of the UPAR in vascular, fibrotic, and coagulation and inflammation pathways and its prior association with non-AIDS events in PWH (25). Future analyses in the main REPRIEVE trial population will relate IL-6 and other inflammatory markers to MACE, cancer endpoints, and other comorbidities to better guide the development of novel therapies for different long-term comorbidities in PWH. It is additionally important to recognize that modifying systemic factors as well as local biological factors at the plaque may both be important and independent of each other. For example, in prior work, we have shown that increased statin-induced expression of collagen-forming genes may help to reduce vulnerable fatty plaque (6), although Procollagen C–endopeptidase enhancer 1 (PCOLCE) was not significantly associated with a reduction in MACE in the current analysis.

In our exploratory enrichment analysis, we observed that many myeloid cell–associated markers were featured prominently in our network analysis and strongly associated with incident MACE. These data further reinforce the prominent role played by myeloid cells in atherogenesis, including initiation and progression with recruitment of monocytes to the foam cells, release of cytokines by engulfed foam cell–resident macrophages, and potential effects of factors to destabilize rupture-prone plaque and increase thrombosis. The inclusion of many of the non-statin-modifiable proteins in enrichment pathways related to myeloid functions of potential effect on atherogenesis suggests that dysregulation of these pathways in PWH may contribute disproportionately to plaque formation and destabilization and should be targeted for future experimentation and therapeutics beyond statin therapy.

These data further beg the question as to whether clonal hematopoiesis driver mutations may be more common in these patients with this particular inflammatory signature (26). Indeed, recent data from our group and others demonstrated a 2-fold increase in clonal hematopoiesis of indeterminate potential among PWH in the Swiss HIV Cohort versus a matched group of participants in the ARIC cohort (27). These data from the current analysis of REPRIEVE add to our knowledge of a potential role of myeloid dysregulation with respect to MACE in low-to-moderate risk PWH on stable ART.

The data in this analysis highlight residual inflammatory pathways that may contribute to MACE, and potentially to other comorbidities, in well-treated PWH on ART. Although some of the pathways identified in the analysis are statin modifiable, while others are not, consistent with data that this trained immunity (more pronounced innate immune responses in previously stimulated cells) persists after statin therapy (28). In contrast, the statin-modifiable pathways appear more likely to prevent ASCVD in PWH through effects on plaque stabilization, lipid lowering, and improved vascular health, which we now show may relate to statin regulation of ANGPTL3 (6).

Of clinical relevance, we demonstrate in cross-validated, time-dependent Cox elastic net machine learning models that the proteins identified in this study may improve the prediction for MACE. The traditional risk scoring model achieved a modest C-index from 0.61, similar to previously published results in this cohort (29). Adding proteins to the model increased the diagnostic performance to a C-index of 0.74. In terms of individual proteins, LAMP3, a lysosome-associated membrane protein that plays a role in various cellular processes, including cell differentiation, autophagy, and immune responses, was most significantly related to a HR well beyond that of ASCVD alone, whereas PON3 was inversely related to a significant degree as a protective pathway. LAMP3 is involved in antigen processing during the immune response and is thought to be linked with the maturation of DCs and potentially a marker of cardiac remodeling, although little is known about how it relates to ASCVD (30, 31). External validation of our findings is needed to assess the additive value of proteomics profiling to identify PWH at increased risk of MACE. Further studies leveraging the data from the larger REPRIEVE cohort will assess these proteins as potentially relevant mechanistic pathways and biomarkers for MACE in PWH.

Our analysis has strengths but also some limitations. The number of events was lower than in the overall REPRIEVE trial because of the smaller size of the mechanistic substudy. Given the limited number of events, our estimates are characterized by a degree of uncertainty, seen in the wide confidence ranges for some of our parameters. Our results are from a population of PWH, therefore, we cannot evaluate whether our findings are specific to this population or may also apply to the general population. Although our results are highly relevant for primary CVD prevention in those PWH with low-to-moderate CVD risk, different results might be seen in those with higher CVD risk. Analyses are ongoing in the overall trial to assess key pathways. In addition, the data that we could analyze were limited to the proteins included in the selected panels, and future studies will address other pathways not fully represented in these panels, for example, to follow up signals we have seen for statins to reduce IL-1β and potentially modulate inflammasome-mediated processes (32). Also, the selective coverage of our targeted proteomics approach may not be optimal for enrichment results. Future analyses within the larger REPRIEVE cohort with extended proteomics coverage will provide further validation of our enrichment results. Furthermore, study-specific NPX values of our Olink proteomics analysis preclude the direct translation to other populations. Future analyses using exact protein concentrations will be needed to define normal values and optimal cutoffs for clinical implementation.

These data demonstrate what we believe to be novel pathways beyond LDL that relate to MACE among PWH. Additional interventions beyond statin therapy targeting these pathways may be useful to decrease MACE in PWH by reducing non-LDL-related lipid mechanisms and residual systemic inflammation. In particular, ANGPTL3, the IL-6 axis, and related pathways may represent the most obvious targets, but heretofore unrecognized pathways including LAMP3 should also be explored as targets. Relevant protein markers may also be used to improve CVD risk prediction in PWH.

## Methods

### Sex as a biological variable.

Sex was not considered a biological variable in this study. Our study examined men and women, without making any distinction between sexes.

### Study design.

Detailed study designs of the REPRIEVE randomized clinical trial (NCT02344290) and the US-based nested mechanistic substudy have been published previously (33, 34). In brief, the REPRIEVE trial enrolled participants with HIV, 40–75 years of age, who received ART for at least 6 months and had low-to-moderate 10-year ASCVD risk scores defined using the 2013 ACC/AHA Pooled Cohort Equation. Participants with prior statin use within 90 days of enrollment or a prior history of ASCVD were excluded (33). At 31 US sites, participants were given the opportunity to co-enroll in the mechanistic substudy and underwent additional blood and imaging analyses (34). Detailed inclusion and exclusion criteria are available in the published study protocols (4, 32). The current analysis is a post hoc analysis of proteomics data, performed to assess the relationship of baseline- and time-updated inflammatory and immune activation biomarkers to the primary clinical trial endpoint of MACE.

### Study population.

Overall, 804 participants were randomized in the mechanistic substudy. In our current analyses, we included all participants who had a valid proteomics measurement before the start of randomized treatment. Proteomics measurements were excluded on the basis of routine proteomics measurement quality control criteria (detailed below). The CONSORT diagram of participant exclusions is shown in Figure 1.

### Proteomics analysis.

Enrollment and 2-year fasting plasma samples stored at –80ºC were used to measure protein levels. The following 3 commercially available multiplex immunoassays were used: Olink Target 96 Cardiovascular III, Immuno-oncology, and Cardiometabolic (all from Thermo Fisher Scientific) to measure 275 unique proteins. We excluded participants for whom complete panels were labeled with warnings indicating potential problems with measurement accuracy. We excluded proteins from the analysis if more than 50% of all samples were lower than the lower limit of detection (Supplemental Figure 1) (35–37). Overall, 255 proteins were evaluated. Proteins are expressed in normalized protein expression values, which are on a log_2_ scale.

### Lipid measurements.

As part of REPRIEVE, fasting blood samples at baseline and 2 years were used to measure LDL-C, HDL-C, total cholesterol, and triglyceride levels. Lipids were measured at Quest Diagnostics.

### Model building assessing the relationship of the protein pathway to MACE.

The goal of the present analysis was to relate the protein pathways to MACE. We first related time-updated protein levels to incident MACE in unadjusted analyses. Subsequently, our analyses adjusted for ASCVD risk score and any use of statins during the follow-up period. Individuals underwent regular study evaluations every 4 months according to the REPRIEVE protocol. Changes to the study drug or other medications, including use of non-study statins, were recorded at each visit. Of note, participants stopping randomized treatment or starting non-study statins continued with REPRIEVE follow-up unchanged, as previously described (4, 5). Using the recorded medication logs, we reconstructed the exact time intervals that participants were either on or off statin therapy. We defined “on statin” time intervals to include use of the randomized statin treatment as per initial treatment assignment to the pitavastatin arm of the trial or receipt of a non-study statin as prescribed by clinical providers. Conversely, time intervals without statin use (i.e., randomized to placebo or holds in randomized pitavastatin) were recorded as “off statin” time. We incorporated these time-updated statin use intervals in our statistical models to adjust for the effects of statin treatment on MACE. This method was used, as our goal was to gain mechanistic insight and thus more accurately assess for actual statin use over longitudinal follow-up in the trial. In a supplemental analysis, we adjusted for ASCVD risk score and randomization, paralleling the intent-to-treat design of the trial.

### Outcome definitions.

The primary endpoint of MACE includes the composite of cardiovascular death, myocardial infarction, hospitalization for unstable angina, coronary, carotid, or peripheral arterial revascularization, transient ischemic attack, stroke, and peripheral arterial ischemia and death due to unknown reason. Detailed definitions are provided in the study protocol (33, 34). We further defined hard MACE as known cardiovascular death, myocardial infarction, and stroke.

### Statistics.

Continuous variables are presented as means and SDs or medians and IQRs, while categorical parameters are shown as counts and percentages. Cox proportional hazards models were used to estimate the association between the most recent protein expression and outcomes. Proteomics measurements (baseline and 2-year) were included as a time-dependent covariate in our models based on the date of the proteomics sampling. Our models were adjusted for baseline 10-year ASCVD risk and time-updated statin use, as previously described. Further analyses were done adding time-dependent LDL-C and triglyceride levels to the model to estimate the protein effect adjusted for current lipid profile. In addition, we performed supplemental analyses, adjusting for initial statin randomization rather than time-updated statin use. HRs are shown per 2-fold higher levels of the protein value. Analysis of the previously identified 7 proteins seen to change with statin therapy used a 5% type 1 error (6). Otherwise, multiple comparisons were adjusted for FDR by the Benjamini and Hochberg method, using a 5% threshold for statistical significance (38). Survival analysis was done using the survival package (v.3.7-0) in R (39).

In sensitivity analyses, we used the psych (version 2.4.6.26) R package to estimate the proportion of randomized statin effects on triglyceride and LDL-C levels mediated through proteins of interest by calculating delta changes in both protein expression and triglyceride levels, defined as the difference between 2-year and baseline values. CIs of the mediated proportion were estimated using the bootstrap method.

Spearman’s or point-biserial correlation was used on baseline proteomics values and lipid and inflammatory markers to evaluate the association between these factors. Protein-protein interaction analysis was done using the Search Tool for the Retrieval of Interacting Genes/Proteins (STRING) database. Confidence-based protein interactions using all available interactions sources were analyzed using a medium confidence threshold (confidence score >0.4). Exploratory Gene Ontology and Reactome functional enrichments were estimated using the whole genome as a background. *P* and FDR values of enrichment are not presented because of the possible bias of our limited set of biologically closely related proteins.

To estimate the additive value of baseline proteins to predict MACE and to evaluate the importance of each proteomics feature, we trained 3 elastic net Cox proportional hazards models with 50% lasso and 50% ridge regularization. Model 1: statin randomization + 10-year ASCVD risk score; model 2: baseline values of proteins associated with MACE; model 3: model 1 + model 2 (including statin randomization, a 10-year ASCVD risk score, and baseline protein levels). We estimated cross-validated, time-dependent inverse, probability-weighted C-index values of an elastic net Cox model to calculate the diagnostic accuracy of our machine learning model over time. We also plotted the cross-validated average HRs per population SD to predict MACE as feature importance values of each parameter for all 3 models, in which at least 1 of the HRs was different from 1 in one of the models. All machine learning survival analyses were done using the scikit-survival (version 0.23.1) python package (40–42).

All statistical calculations were conducted in R (version 4.3.3) and Python (version 3.10.8).

### Study approval.

The REPRIEVE trial protocol was approved by the IRB of the Massachusetts General Brigham Hospital and by the ethics committees of each site (Supplemental Table 7). All participants provided written informed consent. Results are reported in compliance with the Consolidated Standards of Reporting Trials. The trial is registered at ClinicalTrials.gov: NCT02344290.

### Data availability.

All values for the data points in graphs and values behind any reported means are available in the Supporting Data Values file. The Olink data have been added as a supplemental file (baseline values: Supplemental Dataset 1, follow-up values: Supplemental Dataset 2).

## Author contributions

MK performed statistical analysis, interpreted results, and wrote the manuscript. IS interpreted results and helped write the manuscript. MVZ, CJF, JAA, GSB, and JSC participated in the conduct of the trial and reviewed the manuscript draft. SMC reviewed the manuscript draft. MRD and ABL participated in clinical research coordination and reviewed the manuscript draft. CD reviewed the manuscript draft. BF participated in the conduct of the trial and reviewed the manuscript draft. SM collated data, provided statistical support, interpreted results, and reviewed the manuscript draft. CAS reviewed the manuscript draft. MTL participated in the conduct of the trial and reviewed the manuscript draft. PSD designed the study, reviewed the manuscript draft, and provided funding support. HJR provided statistical supervision, interpreted results, and reviewed the manuscript draft. SKG designed the study, interpreted results, wrote the manuscript, and provided funding support. All authors participated in the critical review of the final manuscript and its submission.

## Funding support

This work is the result of NIH funding, in whole or in part, and is subject to the NIH Public Access Policy. Through acceptance of this federal funding, the NIH has been given a right to make the work publicly available in PubMed Central.

- NIH (U01HL123336 and 1UG3HL164285, to the Clinical Coordinating Center).
- NIH (U01HL123339 and 1U24HL164284, to the Data Coordinating Center).
- Kowa Pharmaceuticals America.
- Gilead Sciences.
- ViiV Healthcare.
- NIAID, NIH grants (UM1 AI068636, to the ACTG Leadership and Operations Center; UM1 AI106701, to the ACTG Laboratory Center).
- Nutrition Obesity Research Center at Harvard (P30DK040561, to SKG).
- Intramural Research Program, NIAID, NIH (to IS).

## Supplementary Material

Supplemental data

ICMJE disclosure forms

Supplemental data set 1

Supplemental data set 2

Supporting data values

## Figures and Tables

**Table 3:**
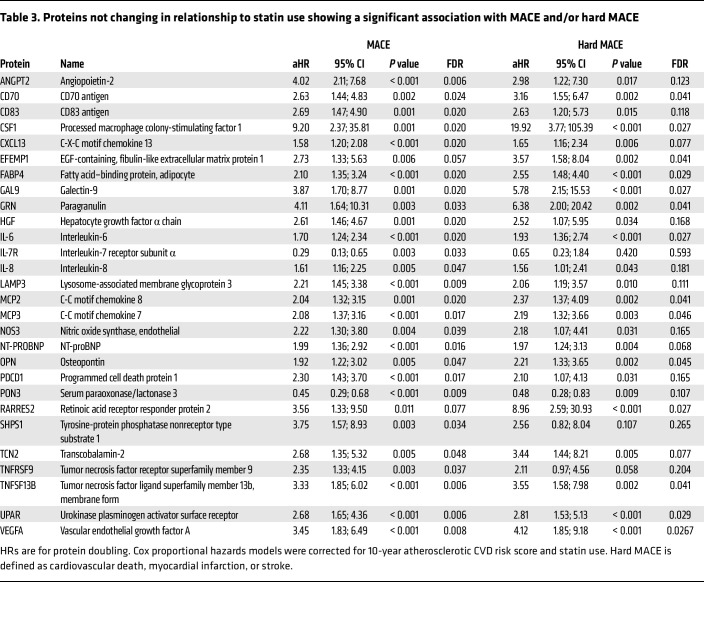
Proteins not changing in relationship to statin use showing a significant association with MACE and/or hard MACE

**Table 2:**
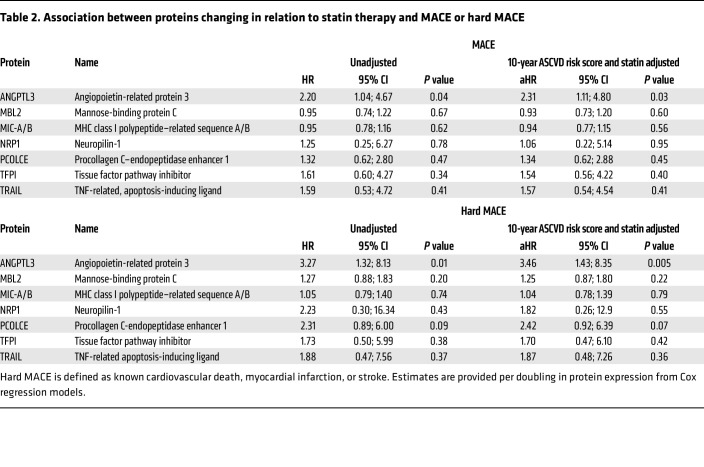
Association between proteins changing in relation to statin therapy and MACE or hard MACE

**Table 1:**
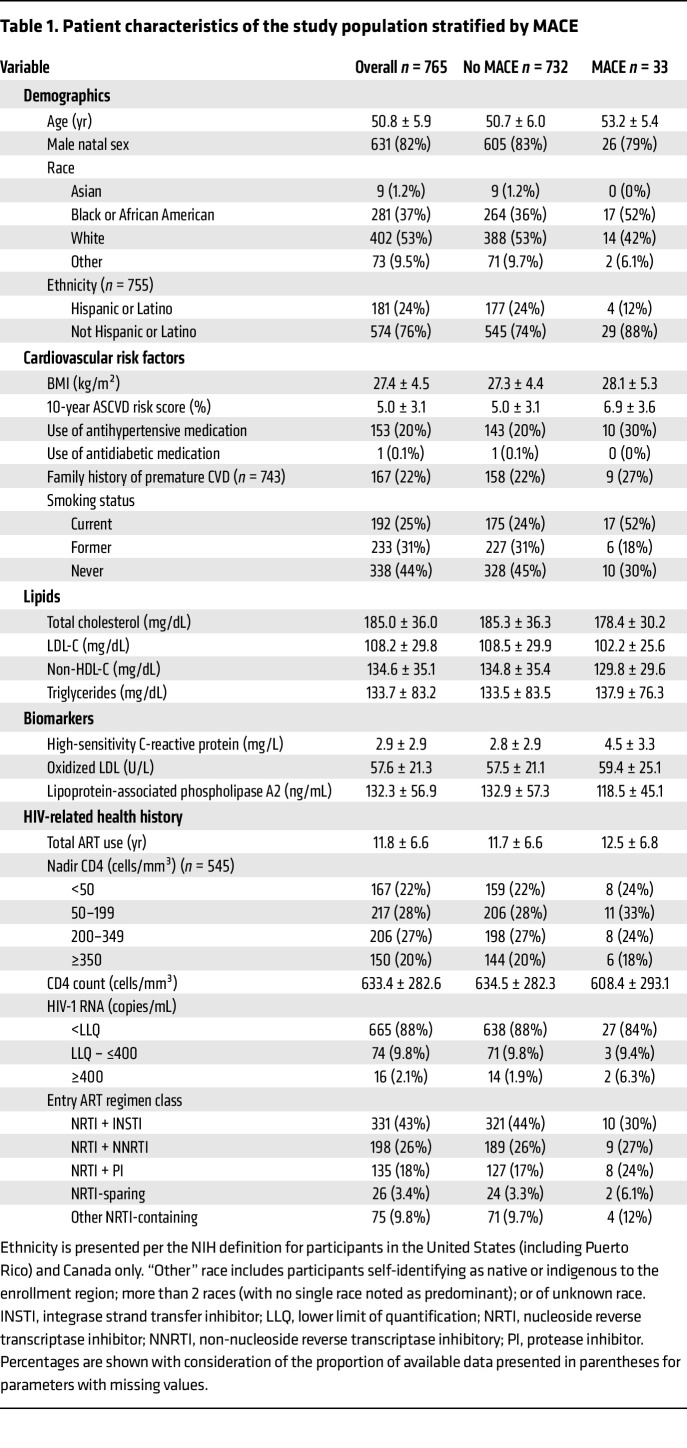
Patient characteristics of the study population stratified by MACE

## Version 1 09/09/2025

In-Press Preview

## Version 2 11/17/2025

Electronic publication
