# Supplementary material for: Statin-dependent and -independent pathways are associated with major adverse cardiovascular events in people with HIV
Source: J Clin Invest. 2025 Sep 9;135(22):e196021. doi: 10.1172/JCI196021 (PMC12618077; doi:10.1172/JCI196021)
Supplement: Supplemental data [file jci-135-196021-s115.pdf]

SUPPLEMENTAL MATERIAL

SUPPLEMENTAL TABLES

Supplemental table 1. Patient characteristics of study population stratified by hard MACE.

Variable	**Overall** N = 765	**No hard MACE** N = 732	**Hard MACE** N = 20
**Demographics**			
Age (years)	50.8 ± 5.9	50.7 ± 5.9	54.4 ± 5.6
Male natal sex	631 (82%)	616 (83%)	15 (75%)
Race			
Asian	9 (1.2%)	9 (1.2%)	0 (0%)
Black or African American	281 (37%)	271 (36%)	10 (50%)
White	402 (53%)	393 (53%)	9 (45%)
Other	73 (9.5%)	72 (9.7%)	1 (5.0%)
Ethnicity (n=755)			
Hispanic or Latino	181 (24%)	178 (24%)	3 (15%)
Not Hispanic or Latino	574 (75%)	557 (75%)	17 (85%)
**Cardiovascular risk factors**			
BMI (kg/m²)	27.4 ± 4.5	27.3 ± 4.4	28.1 ± 5.5
10-year ASCVD risk score (%)	5.0 ± 3.1	5.0 ± 3.1	7.0 ± 3.9
Use of antihypertensive medication	153 (20%)	147 (20%)	6 (30%)
Use of antidiabetic medication	1 (0.1%)	1 (0.1%)	0 (0%)
Family history of premature CVD (n=743)	167 (22%)	162 (22%)	5 (25%)
Smoking status			
Current	192 (25%)	181 (24%)	11 (55%)
Former	233 (31%)	230 (31%)	3 (15%)
Never	338 (44%)	332 (45%)	6 (30%)
**Lipids**			
Total cholesterol (mg/dL)	185.0 ± 36.0	185.1 ± 36.2	180.4 ± 29.4
LDL-C (mg/dL)	108.2 ± 29.8	108.3 ± 29.9	103.3 ± 22.9
Non-HDL-C (mg/dL)	134.6 ± 35.1	134.7 ± 35.3	131.7 ± 30.5
Triglycerides (mg/dL)	133.7 ± 83.2	133.4 ± 83.1	142.0 ± 86.3
**Biomarkers**			
High-sensitivity C-reactive protein (mg/L)	2.9 ± 2.9	2.9 ± 2.9	5.1 ± 3.3
Oxidized low-density lipoprotein (U/L)	57.6 ± 21.3	57.4 ± 21.1	65.0 ± 25.7
Lipoprotein-associated phospholipase A2 (ng/mL)	132.3 ± 56.9	132.9 ± 57.3	109.9 ± 35.8
**HIV-related health history**			
Total ART use (years)	11.8 ± 6.6	11.8 ± 6.6	11.3 ± 6.8
Nadir CD4 (cells/mm³) (n=545)			
<50	167 (22%)	163 (22%)	4 (20%)
50-199	217 (28%)	208 (28%)	9 (45%)
200-349	206 (27%)	203 (27%)	3 (15%)
350+	150 (20%)	146 (20%)	4 (20%)
CD4 count (cells/mm³)	633.4 ± 282.6	633.6 ± 281.2	626.0 ± 337.1
HIV-1 RNA (copies/mL)			
<LLQ	665 (88%)	648 (88%)	17 (89%)
LLQ -< 400	74 (9.8%)	73 (9.9%)	1 (5.3%)
400+	16 (2.1%)	15 (2.0%)	1 (5.3%)
Entry ART regimen class			
NRTI + INSTI	331 (43%)	324 (43%)	7 (35%)
NRTI + NNRTI	198 (26%)	191 (26%)	7 (35%)
NRTI + PI	135 (18%)	131 (18%)	4 (20%)
NRTI-sparing	26 (3.4%)	25 (3.4%)	1 (5.0%)
Other NRTI-containing	75 (9.8%)	74 (9.9%)	1 (5.0%)

Ethnicity presented per NIH definition for participants in US (including Puerto Rico) and Canada only. 'Other' race includes participants self-identifying as: native or indigenous to the enrollment region; more than one race (with no single race noted as predominant); or of unknown race. Hard MACE was defined as: cardiovascular deaths, myocardial infarction or stroke.

Abbreviations: ART, antiretroviral therapy; ASCVD, atherosclerotic cardiovascular disease; BMI, body mass index; CVD, cardiovascular disease; HDL-C, high-density lipoprotein cholesterol; HIV, human immunodeficiency virus; INSTI, integrase strand transfer inhibitor; LDL-C, low-density lipoprotein cholesterol; LLQ, lower limit of quantification; MACE, major adverse cardiac event; NRTI, nucleoside reverse transcriptase inhibitor; NNRTI, nonnucleoside reverse transcriptase inhibitory; PI, protease inhibitor.

Percentages are presented considering the proportion of available data presented in parenthesis for parameters with missing values.

Supplemental table 2. Association between proteins changing in relation to statin therapy and MACE and hard MACE – adjusting models using statin randomization.

MACE

Protein	Name	10-year ASCVD risk score, statin randomization adjusted		
		**aHR**	**95% CI**	**p**
ANGPTL3	angiopoietin-related protein 3	2.28	1.08; 4.84	0.03
MBL2	mannose-binding protein C	0.94	0.73; 1.2	0.60
MIC-A/B	MHC class I polypeptide-related sequence A/B	0.94	0.77; 1.16	0.59
NRP1	neuropilin-1	1.16	0.24; 5.76	0.85
PCOLCE	procollagen C-endopeptidase enhancer 1	1.42	0.66; 3.08	0.37
TFPI	tissue factor pathway inhibitor	1.46	0.53; 4.03	0.46
TRAIL	TNF-related apoptosis-inducing ligand	1.51	0.52; 4.39	0.45

Hard MACE

Protein	Name	10-year ASCVD risk score, statin randomization adjusted		
		**aHR**	**95% CI**	**p**
ANGPTL3	angiopoietin-related protein 3	3.42	1.40; 8.40	0.007
MBL2	mannose-binding protein C	1.25	0.87; 1.80	0.22
MIC-A/B	MHC class I polypeptide-related sequence A/B	1.05	0.78; 1.40	0.77
NRP1	neuropilin-1	1.98	0.28; 14.16	0.50
PCOLCE	procollagen C-endopeptidase enhancer 1	2.57	0.98; 6.76	0.06
TFPI	tissue factor pathway inhibitor	1.62	0.45; 5.82	0.46
TRAIL	TNF-related apoptosis-inducing ligand	1.79	0.46; 6.98	0.40

Hard MACE was defined as: cardiovascular death, myocardial infarction or stroke. Estimates are provided per doubling in protein expression from Cox regression models.
Abbreviations: aHR, adjusted hazard ratio; CI, confidence interval

Supplemental table 3. Association between proteins changing in relation to statin therapy and MACE and hard MACE – additional adjustments to lipid levels.

MACE

Protein	Name	10-year ASCVD risk score, statin and LDL-C adjusted			10-year ASCVD risk score, statin and triglyceride adjusted			10-year ASCVD risk score, statin, LDL-Cand triglyceride adjusted		
		**aHR**	**95% CI**	**p**	**aHR**	**95% CI**	**p**	**aHR**	**95% CI**	**p**
ANGPTL3	angiopoietin-related protein 3	2.30	1.09; 4.87	0.03	2.12	0.99; 4.53	0.05	2.30	1.08; 4.87	0.03
MBL2	mannose-binding protein C	0.94	0.73; 1.22	0.65	0.94	0.73; 1.21	0.63	0.94	0.73; 1.21	0.63
MIC-A/B	MHC class I polypeptide-related sequence A/B	0.92	0.75; 1.12	0.40	0.92	0.75; 1.13	0.45	0.92	0.75; 1.12	0.40
NRP1	neuropilin-1	0.77	0.15; 3.87	0.75	0.79	0.16; 4.06	0.78	0.79	0.15; 4.04	0.78
PCOLCE	procollagen C-endopeptidase enhancer 1	1.36	0.62; 2.96	0.44	1.42	0.65; 3.11	0.38	1.35	0.62; 2.95	0.45
TFPI	tissue factor pathway inhibitor	2.60	0.86; 7.83	0.09	1.74	0.62; 4.90	0.29	2.59	0.85; 7.95	0.09
TRAIL	TNF-related apoptosis-inducing ligand	1.71	0.59; 4.98	0.32	1.62	0.55; 4.75	0.38	1.71	0.59; 4.97	0.33

Hard MACE

Protein	Name	10-year ASCVD risk score, statin and LDL-C adjusted			10-year ASCVD risk score, statin and triglyceride adjusted			10-year ASCVD risk score, statin, LDL-Cand triglyceride adjusted		
		**aHR**	**95% CI**	**p**	**aHR**	**95% CI**	**p**	**aHR**	**95% CI**	**p**
ANGPTL3	angiopoietin-related protein 3	3.83	1.65; 8.89	0.001	3.47	1.43; 8.37	0.005	3.84	1.64; 9.00	0.002
MBL2	mannose-binding protein C	1.25	0.87; 1.80	0.22	1.26	0.87; 1.8	0.22	1.25	0.87; 1.80	0.23
MIC-A/B	MHC class I polypeptide-related sequence A/B	1.03	0.77; 1.38	0.83	1.04	0.78; 1.39	0.78	1.03	0.77; 1.38	0.84
NRP1	neuropilin-1	1.74	0.25; 12.22	0.58	1.98	0.27; 14.37	0.50	1.93	0.27; 13.93	0.51
PCOLCE	procollagen C-endopeptidase enhancer 1	2.27	0.86; 6.02	0.10	2.39	0.9; 6.33	0.08	2.24	0.85; 5.94	0.10
TFPI	tissue factor pathway inhibitor	2.71	0.69; 10.67	0.16	1.68	0.46; 6.09	0.43	2.59	0.65; 10.43	0.18
TRAIL	TNF-related apoptosis-inducing ligand	2.02	0.53; 7.67	0.30	1.89	0.49; 7.28	0.36	2.02	0.53; 7.68	0.30

Hard MACE was defined as: cardiovascular death, myocardial infarction or stroke. Estimates are provided per doubling in protein expression from Cox regression models.
Abbreviations: aHR, adjusted hazard ratio; CI, confidence interval

Supplemental table 4. Association between proteomic markers and MACE.

Protein	Name	Olink ID	UniProt	aHR	95% lower CI	95% higher CI	p	FDR
ADA	Adenosine deaminase	OID00775	P00813	1.78	1.07	2.96	0.02659	0.11988
ALCAM	CD166 antigen	OID00572	Q13740	2.27	1.04	4.94	0.03978	0.15274
ANG	Angiogenin	OID01226	P03950	0.69	0.34	1.40	0.29932	0.51195
ANGPT1	Angiopoietin-1	OID00760	Q15389	1.45	1.01	2.07	0.04309	0.15715
ANGPT2	Angiopoietin-2	OID00822	O15123	4.02	2.11	7.68	0.00002	0.00595
AOC3	Membrane primary amine oxidase	OID01294	Q16853	0.97	0.34	2.75	0.95898	0.97783
APN	Aminopeptidase N	OID00611	P15144	0.79	0.24	2.52	0.68518	0.83939
APOM	Apolipoprotein M	OID01221	O95445	0.42	0.19	0.92	0.02980	0.13199
ARG1	Arginase-1	OID00815	P05089	0.84	0.55	1.30	0.44279	0.63474
AXL	Tyrosine-protein kinase receptor UFO	OID00612	P30530	1.01	0.38	2.66	0.98842	0.98842
AZU1	Azurocidin	OID00597	P20160	1.31	0.87	1.98	0.20230	0.41458
BLM HYDROLASE	Bleomycin hydrolase	OID00581	Q13867	1.11	0.59	2.09	0.73584	0.84879
C1QTNF1	Complement C1q tumor necrosis factor-related protein 1	OID01301	Q9BXJ1	1.00	0.59	1.68	0.98634	0.98842
C2	Complement C2a fragment	OID01233	P06681	1.21	0.45	3.26	0.70166	0.83939
CA1	Carbonic anhydrase 1	OID01223	P00915	0.97	0.65	1.46	0.90211	0.94038
CA3	Carbonic anhydrase 3	OID01235	P07451	1.15	0.62	2.15	0.64904	0.83399
CA4	Carbonic anhydrase 4	OID01261	P22748	0.83	0.25	2.76	0.75709	0.85735
CAIX	Carbonic anhydrase 9	OID00773	Q16790	1.03	0.51	2.05	0.94317	0.96655
CASP3	Caspase-3 subunit p12	OID00630	P42574	1.22	0.99	1.51	0.06157	0.19087
CASP8	Caspase-8 subunit p10	OID00827	Q14790	1.46	1.01	2.10	0.04586	0.15965
CCL14	C-C motif chemokine 14	OID01292	Q16627	1.43	0.66	3.08	0.36568	0.57397
CCL15	C-C motif chemokine 15	OID00629	Q16663	1.17	0.66	2.09	0.58464	0.77525
CCL16	C-C motif chemokine 16	OID00654	O15467	1.12	0.72	1.74	0.61190	0.79869
CCL17	C-C motif chemokine 17	OID00821	Q92583	1.38	1.04	1.83	0.02469	0.11665
CCL18	C-C motif chemokine 18	OID01276	P55774	1.43	0.94	2.18	0.09917	0.26685
CCL19	C-C motif chemokine 19	OID00794	Q99731	1.48	1.01	2.18	0.04413	0.15861
CCL20	C-C motif chemokine 20	OID00837	P78556	1.08	0.79	1.47	0.62575	0.81249
CCL23	C-C motif chemokine 23	OID00811	P55773	1.47	0.79	2.73	0.21840	0.42916
CCL24	C-C motif chemokine 24	OID00592	O00175	0.82	0.55	1.23	0.34826	0.56821
CCL3	C-C motif chemokine 3	OID00813	P10147	1.25	1.01	1.54	0.03604	0.14897
CCL4	C-C motif chemokine 4	OID00796	P13236	1.29	0.82	2.05	0.26889	0.48323
CCL5	C-C motif chemokine 5	OID01246	P13501	1.19	1.00	1.41	0.04835	0.15988
CD163	Scavenger receptor cysteine-rich type 1 protein M130	OID00577	Q86VB7	1.11	0.59	2.10	0.74050	0.85003
CD244	Natural killer cell receptor 2B4	OID00758	Q9BZW8	1.48	0.91	2.41	0.11044	0.28237
CD27	CD27 antigen	OID00800	P26842	2.89	1.30	6.40	0.00893	0.06918
CD4	T-cell surface glycoprotein CD4	OID00776	P01730	1.42	0.65	3.08	0.38156	0.58449
CD40	Tumor necrosis factor receptor superfamily member 5	OID00781	P25942	2.01	1.16	3.48	0.01233	0.08048
CD40L	CD40 ligand, membrane form	OID00756	P29965	1.29	1.05	1.59	0.01689	0.09308
CD46	Membrane cofactor protein	OID01251	P15529	1.23	0.95	1.61	0.12118	0.29755
CD5	T-cell surface glycoprotein CD5	OID00812	P06127	2.23	1.01	4.89	0.04635	0.15965
CD59	CD59 glycoprotein	OID01248	P13987	1.10	0.53	2.27	0.80338	0.87610
CD70	CD70 antigen	OID00808	P32970	2.63	1.44	4.83	0.00176	0.02432
CD83	CD83 antigen	OID00841	Q01151	2.69	1.47	4.90	0.00129	0.01988
CD8A	T-cell surface glycoprotein CD8 alpha chain	OID00772	P01732	1.23	0.77	1.95	0.38180	0.58449
CD93	Complement component C1q receptor	OID00639	Q9NPY3	1.79	0.69	4.67	0.23266	0.44384
CDH1	Cadherin-1	OID01245	P12830	1.35	0.61	2.97	0.45462	0.64040
CDH5	Cadherin-5	OID00587	P33151	1.02	0.38	2.71	0.96994	0.97783
CES1	Liver carboxylesterase 1	OID01263	P23141	1.03	0.66	1.62	0.89537	0.94038
CFHR5	Complement factor H-related protein 5	OID01302	Q9BXR6	1.10	0.52	2.30	0.80289	0.87610
CHI3L1	Chitinase-3-like protein 1	OID00633	P36222	1.25	0.94	1.66	0.12948	0.31456
CHIT1	Chitotriosidase-1	OID00605	Q13231	0.91	0.71	1.16	0.43957	0.63379
CHL1	Processed neural cell adhesion molecule L1-like protein	OID01216	O00533	1.18	0.36	3.92	0.78330	0.87567
CNDP1	Carnosine dipeptidase 1	OID01299	Q96KN2	0.78	0.51	1.21	0.27237	0.48595

Protein	Name	Olink ID	UniProt	aHR	95% lower CI	95% higher CI	p	FDR
CNTN1	Contactin-1	OID00586	Q12860	0.51	0.17	1.56	0.23768	0.44995
COL18A1	Collagen alpha-1(XVIII) chain	OID01271	P39060	1.52	0.57	4.05	0.40657	0.61264
COL1A1	Collagen alpha-1(I) chain	OID00641	P02452	2.89	1.27	6.59	0.01153	0.07726
COMP	Cartilage oligomeric matrix protein	OID01274	P49747	0.90	0.45	1.81	0.76681	0.86440
CPA1	Carboxypeptidase A1	OID00624	P15085	1.08	0.70	1.67	0.71754	0.83939
CPB1	Carboxypeptidase B1	OID00632	P15086	1.11	0.72	1.72	0.64361	0.83133
CR2	Complement receptor type 2	OID01258	P20023	1.06	0.55	2.05	0.86543	0.92115
CRTAC1	Cartilage acidic protein 1	OID01304	Q9NQ79	0.58	0.27	1.26	0.16831	0.36616
CRTAM	Cytotoxic and regulatory T-cell molecule	OID00766	O95727	1.66	1.03	2.67	0.03773	0.15274
CSF1	Processed macrophage colony-stimulating factor 1	OID00843	P09603	9.20	2.37	35.81	0.00136	0.01988
CST3	Cystatin-C	OID01225	P01034	2.01	0.87	4.61	0.10115	0.26685
CSTB	Cystatin-B	OID00575	P04080	1.70	1.15	2.51	0.00781	0.06675
CTSD	Cathepsin D heavy chain	OID00622	P07339	1.41	0.77	2.59	0.26652	0.48246
CTSZ	Cathepsin Z	OID00643	Q9UBR2	1.81	0.72	4.51	0.20536	0.41458
CX3CL1	Processed fractalkine	OID00806	P78423	1.83	0.69	4.82	0.22321	0.43037
CXCL1	Growth-regulated alpha protein	OID00786	P09341	1.29	0.96	1.71	0.08761	0.24973
CXCL10	C-X-C motif chemokine 10	OID00807	P02778	1.30	0.93	1.82	0.13064	0.31456
CXCL11	C-X-C motif chemokine 11	OID00767	O14625	1.21	0.92	1.60	0.17840	0.38473
CXCL12	Stromal cell-derived factor 1	OID00824	P48061	3.07	1.39	6.77	0.00556	0.05110
CXCL13	C-X-C motif chemokine 13	OID00830	O43927	1.58	1.20	2.08	0.00098	0.01988
CXCL16	C-X-C motif chemokine 16	OID00601	Q9H2A7	2.32	0.81	6.62	0.11754	0.29151
CXCL5	C-X-C motif chemokine 5	OID00801	P42830	1.39	1.08	1.79	0.01059	0.07726
CXCL9	C-X-C motif chemokine 9	OID00771	Q07325	1.30	0.91	1.87	0.15224	0.34958
DCN	Decorin	OID00817	P07585	0.92	0.46	1.87	0.82755	0.89621
DLK1	Protein delta homolog 1	OID00598	P80370	1.31	0.77	2.20	0.31640	0.52663
DPP4	Dipeptidyl peptidase 4 membrane form	OID01266	P27487	0.61	0.24	1.57	0.30394	0.51461
EFEMP1	EGF-containing fibulin-like extracellular matrix protein 1	OID01281	Q12805	2.73	1.33	5.63	0.00648	0.05740
EGF	Pro-epidermal growth factor	OID00759	P01133	1.24	1.00	1.54	0.05318	0.17352
EGFR	Epidermal growth factor receptor	OID00637	P00533	0.39	0.07	2.16	0.28351	0.50032
ENG	Endoglin	OID01254	P17813	1.31	0.58	2.97	0.52056	0.70792
EPCAM	Epithelial cell adhesion molecule	OID00610	P16422	0.92	0.62	1.35	0.66877	0.83939
EPHB4	Ephrin type-B receptor 4	OID00569	P54760	2.28	0.72	7.26	0.16364	0.36062
F11	Coagulation factor XIa heavy chain	OID01227	P03951	0.67	0.29	1.58	0.36509	0.57397
F7	Coagulation factor VII	OID01239	P08709	1.10	0.36	3.34	0.86453	0.92115
FABP4	Fatty acid-binding protein, adipocyte	OID00589	P15090	2.10	1.35	3.24	0.00089	0.01988
FAS	Tumor necrosis factor receptor superfamily member 6	OID00615	P25445	1.57	0.83	2.96	0.16429	0.36062
FASLG	Tumor necrosis factor ligand superfamily member 6, membrane form	OID00792	P48023	1.05	0.48	2.30	0.90246	0.94038
FCGR2A	Low affinity immunoglobulin gamma Fc region receptor II-a	OID01244	P12318	1.23	0.71	2.12	0.45706	0.64040
FCGR3B	Low affinity immunoglobulin gamma Fc region receptor III-B	OID01219	O75015	1.55	0.79	3.04	0.20298	0.41458
FCN2	Ficolin-2	OID01290	Q15485	1.01	0.59	1.72	0.96670	0.97783
FETUB	Fetuin-B	OID01305	Q9UGM5	0.97	0.49	1.93	0.92631	0.95321
FGF2	Fibroblast growth factor 2	OID00770	P09038	1.47	1.06	2.04	0.02055	0.10617
GAL1	Galectin-1	OID00798	P09382	1.30	0.36	4.67	0.69205	0.83939
GAL3	Galectin-3	OID00578	P17931	1.18	0.49	2.85	0.70502	0.83939
GAL4	Galectin-4	OID00626	P56470	1.08	0.62	1.88	0.78740	0.87567
GAL9	Galectin-9	OID00779	O00182	3.87	1.70	8.77	0.00122	0.01988
GAS6	Growth arrest-specific protein 6	OID01286	Q14393	2.37	0.90	6.20	0.07910	0.22809
GDF15	Growth/differentiation factor 15	OID00595	Q99988	1.80	1.10	2.93	0.01887	0.09957
GNLY	Granulysin	OID01262	P22749	0.89	0.50	1.58	0.69594	0.83939
GP1BA	Platelet glycoprotein Ib alpha chain	OID01234	P07359	1.24	0.98	1.57	0.07578	0.22109

Protein	Name	Olink ID	UniProt	aHR	95% lower CI	95% higher CI	p	FDR
GP6	Platelet glycoprotein VI	OID05026	Q9HCN6	1.37	0.95	1.97	0.09165	0.25537
GRN	Paragranulin	OID00579	P28799	4.11	1.64	10.31	0.00257	0.03289
GZMA	Granzyme A	OID00804	P12544	1.33	0.89	2.00	0.16432	0.36062
GZMB	Granzyme B	OID00840	P10144	1.19	0.90	1.58	0.22386	0.43037
GZMH	Granzyme H	OID00783	P20718	1.17	0.88	1.55	0.28647	0.50032
HGF	Hepatocyte growth factor alpha chain	OID00803	P14210	2.61	1.46	4.67	0.00127	0.01988
HO1	Heme oxygenase 1	OID00805	P09601	0.67	0.31	1.41	0.28968	0.50238
ICAM2	Intercellular adhesion molecule 2	OID00646	P13598	1.17	0.45	3.01	0.74681	0.85003
ICAM1	Intercellular adhesion molecule 1	OID01230	P05362	2.91	1.28	6.66	0.01117	0.07726
ICAM3	Intercellular adhesion molecule 3	OID01267	P32942	1.34	0.63	2.88	0.44684	0.63688
ICOSLG	Ig-like domain-containing protein	OID00828	O75144	0.30	0.07	1.23	0.09382	0.25853
IFNG	Interferon gamma	OID05552	P01579	1.22	0.92	1.60	0.16169	0.36062
IGFBP1	Insulin-like growth factor-binding protein 1	OID00604	P08833	1.02	0.75	1.39	0.88450	0.93741
IGFBP2	Insulin-like growth factor-binding protein 2	OID00650	P18065	1.57	1.02	2.42	0.04159	0.15626
IGFBP7	Insulin-like growth factor-binding protein 7	OID00638	Q16270	2.62	1.14	6.03	0.02382	0.11582
IGFBP3	Insulin-like growth factor-binding protein 3	OID01255	P17936	0.76	0.39	1.48	0.42238	0.62282
IGFBP6	Insulin-like growth factor-binding protein 6	OID01264	P24592	0.82	0.32	2.10	0.67442	0.83939
IGLC2	Ig lambda-2 chain C regions	OID01240	P0DOY2	1.81	1.03	3.17	0.03838	0.15274
IL17RA	Interleukin-17 receptor A	OID00566	Q96F46	0.99	0.55	1.77	0.96315	0.97783
IL18BP	Interleukin-18-binding protein	OID00640	O95998	2.54	1.14	5.66	0.02230	0.11060
IL1RT1	Interleukin-1 receptor type 1, membrane form	OID00613	P14778	1.26	0.38	4.19	0.70823	0.83939
IL1RT2	Interleukin-1 receptor type 2, membrane form	OID00627	P27930	0.56	0.21	1.53	0.26046	0.48170
IL6RA	Interleukin-6 receptor subunit alpha	OID00602	P08887	2.99	1.24	7.22	0.01466	0.08659
IL10	Interleukin-10	OID00809	P22301	1.04	0.61	1.77	0.89134	0.94038
IL12	Interleukin-12 subunit alpha and beta	OID00842	P29459, P29460	1.39	0.88	2.20	0.15485	0.35232
IL12RB1	Interleukin-12 receptor subunit beta-1	OID00835	P42701	1.57	0.77	3.17	0.21344	0.42501
IL15	Interleukin-15	OID05551	P40933	1.80	0.65	4.98	0.25775	0.48062
IL18	Interleukin-18	OID00782	Q14116	1.71	1.01	2.91	0.04786	0.15988
IL2RA	Interleukin-2 receptor subunit alpha	OID00570	P01589	2.03	1.06	3.89	0.03281	0.14137
IL6	Interleukin-6	OID00763	P05231	1.70	1.24	2.34	0.00099	0.01988
IL7	Interleukin-7	OID00761	P13232	1.49	1.08	2.05	0.01461	0.08659
IL7R	Interleukin-7 receptor subunit alpha	OID01253	P16871	0.29	0.13	0.65	0.00265	0.03289
IL8	Interleukin-8	OID00752	P10145	1.61	1.16	2.25	0.00477	0.04733
ITGB2	Integrin beta-2	OID00565	P05107	1.53	0.63	3.74	0.34542	0.56732
JAMA	Junctional adhesion molecule A	OID00625	Q9Y624	1.27	0.98	1.63	0.06992	0.20891
KIR3DL1	Killer cell immunoglobulin-like receptor 3DL1	OID05550	P43629	1.33	0.93	1.89	0.11744	0.29151
KIT	Mast/stem cell growth factor receptor Kit	OID01241	P10721	0.91	0.35	2.37	0.85303	0.91580
KLK6	Kallikrein-6	OID00647	Q92876	0.86	0.33	2.20	0.74720	0.85003
KLRD1	Natural killer cells antigen CD94	OID00839	Q13241	1.03	0.62	1.70	0.91029	0.94063
LAG3	Secreted lymphocyte activation gene 3 protein	OID05553	P18627	1.60	0.97	2.63	0.06418	0.19650
LAMP3	Lysosome-associated membrane glycoprotein 3	OID00826	Q9UQV4	2.21	1.45	3.38	0.00022	0.00930
LAP TGFB1	Transforming growth factor beta-1 proprotein	OID00785	P01137	1.70	1.12	2.59	0.01276	0.08111
LDL RECEPTOR	Low-density lipoprotein receptor	OID00564	P01130	0.77	0.41	1.42	0.39666	0.60350

Protein	Name	Olink ID	UniProt	aHR	95% lower CI	95% higher CI	p	FDR
LILRB1	Leukocyte immunoglobulin-like receptor subfamily B member 1	OID01297	Q8NHL6	1.55	0.77	3.12	0.21977	0.42916
LILRB2	Leukocyte immunoglobulin-like receptor subfamily B member 2	OID01296	Q8N423	1.44	0.88	2.35	0.14915	0.34811
LILRB5	Leukocyte immunoglobulin-like receptor subfamily B member 5	OID01220	O75023	1.07	0.63	1.82	0.80457	0.87610
LTBR	Tumor necrosis factor receptor superfamily member 3	OID00583	P36941	1.47	0.51	4.25	0.47478	0.66059
LYVE1	Lymphatic vessel endothelial hyaluronic acid receptor 1	OID01307	Q9Y5Y7	0.84	0.36	1.98	0.69491	0.83939
MB	Myoglobin	OID00616	P02144	1.45	0.88	2.38	0.14830	0.34811
MCP1	C-C motif chemokine 2	OID00576	P13500	1.68	0.74	3.83	0.21422	0.42501
MCP2	C-C motif chemokine 8	OID00795	P80075	2.04	1.32	3.15	0.00125	0.01988
MCP3	C-C motif chemokine 7	OID00755	P80098	2.08	1.37	3.16	0.00061	0.01691
MCP4	C-C motif chemokine 13, medium chain	OID00768	Q99616	1.67	1.14	2.44	0.00882	0.06918
MEGF9	Multiple epidermal growth factor-like domains protein 9	OID01303	Q9H1U4	0.68	0.22	2.09	0.49906	0.68760
MEPE	Matrix extracellular phosphoglycoprotein	OID00132	Q9NQ76	0.67	0.28	1.61	0.37277	0.57780
MET	Hepatocyte growth factor receptor	OID01238	P08581	1.78	0.36	8.70	0.47680	0.66059
MFAP5	Microfibrillar-associated protein 5	OID01285	Q13361	1.18	0.34	4.17	0.79520	0.87610
MMP2	72 kDa type IV collagenase	OID00614	P08253	1.43	0.49	4.16	0.50851	0.69674
MMP3	Stromelysin-1	OID00644	P08254	0.71	0.40	1.29	0.26221	0.48170
MMP9	67 kDa matrix metalloproteinase-9	OID00568	P14780	1.09	0.68	1.74	0.71337	0.83939
MMP12	Macrophage metalloelastase	OID00829	P39900	1.27	0.83	1.94	0.26455	0.48242
MMP7	Matrilysin	OID00814	P09237	1.94	1.09	3.45	0.02493	0.11665
MPO	Myeloperoxidase heavy chain	OID00600	P05164	0.79	0.33	1.88	0.58974	0.77525
MUC16	Mucin-16	OID05549	Q8WXI7	0.81	0.50	1.33	0.41018	0.61280
NCAM1	Neural cell adhesion molecule 1	OID01247	P13591	0.36	0.13	1.02	0.05459	0.17583
NCR1	Natural cytotoxicity triggering receptor 1	OID00816	O76036	2.23	1.01	4.93	0.04623	0.15965
NID1	Nidogen-1	OID01250	P14543	1.32	0.96	1.82	0.08896	0.25071
NOS3	Nitric oxide synthase, endothelial	OID00777	P29474	2.22	1.30	3.80	0.00363	0.03912
NOTCH 3	Neurogenic locus notch homolog protein 3	OID00584	Q9UM47	1.80	0.73	4.44	0.19970	0.41458
NOTCH1	Neurogenic locus notch homolog protein 1	OID01273	P46531	1.60	0.32	7.92	0.56799	0.76556
NT-PROBNP	NT-proBNP	OID00131	NA	1.99	1.36	2.92	0.00044	0.01566
OPG	Tumor necrosis factor receptor superfamily member 11B	OID00571	O00300	1.98	0.88	4.49	0.10027	0.26685
OPN	Osteopontin	OID00621	P10451	1.92	1.22	3.02	0.00458	0.04733
OSMR	Oncostatin-M-specific receptor subunit beta	OID01300	Q99650	5.38	1.14	25.42	0.03347	0.14137
PAI	Plasminogen activator inhibitor 1	OID00591	P05121	1.35	0.93	1.95	0.11433	0.28932
PAM	Peptidyl-alpha-hydroxyglycine alpha-amidating lyase	OID01256	P19021	1.62	0.50	5.29	0.42442	0.62282
PCSK9	Proprotein convertase subtilisin/kexin type 9	OID00619	Q8NBP7	2.02	0.69	5.92	0.19849	0.41458
PDL1	Programmed cell death 1 ligand 1	OID00799	Q9NZQ7	1.99	1.08	3.64	0.02635	0.11988
PDL2	Programmed cell death 1 ligand 2	OID00831	Q9BQ51	1.14	0.46	2.82	0.78416	0.87567
PDCD1	Programmed cell death protein 1	OID00791	Q15116	2.30	1.43	3.70	0.00061	0.01691
PDGF SUBUNIT A	Platelet-derived growth factor subunit A	OID00648	P04085	1.42	1.02	1.98	0.03987	0.15274
PDGF SUBUNIT B	Platelet-derived growth factor subunit B	OID00790	P01127	1.61	1.00	2.57	0.04765	0.15988
PECAM1	Platelet endothelial cell adhesion molecule	OID00652	P16284	1.39	0.99	1.96	0.05981	0.18775
PGF	Placenta growth factor	OID00762	P49763	1.60	0.50	5.14	0.42831	0.62483

Protein	Name	Olink ID	UniProt	aHR	95% lower CI	95% higher CI	p	FDR
PGLYRP1	Peptidoglycan recognition protein 1	OID00623	O75594	1.45	0.73	2.89	0.28527	0.50032
PI3	Elafin	OID00609	P19957	0.93	0.51	1.67	0.80545	0.87610
PLC	Basement membrane-specific heparan sulfate proteoglycan core protein	OID00582	P98160	1.62	0.52	5.07	0.40760	0.61264
PLTP	Phospholipid transfer protein	OID01275	P55058	0.76	0.20	2.87	0.68207	0.83939
PLXNB2	Plexin-B2	OID01218	O15031	1.61	0.64	4.04	0.31381	0.52584
PON3	Serum paraoxonase/lactonase 3	OID00642	Q15166	0.45	0.29	0.68	0.00018	0.00884
PROC	Vitamin K-dependent protein C heavy chain	OID01228	P04070	0.40	0.19	0.83	0.01349	0.08366
PRSS2	Trypsin-2	OID01236	P07478	1.18	0.65	2.13	0.59081	0.77525
PRTN3	Myeloblastin	OID00618	P24158	1.41	0.73	2.69	0.30503	0.51461
PSPD	Pulmonary surfactant-associated protein D	OID00608	P35247	1.29	0.76	2.20	0.34427	0.56732
PTPRS	Receptor-type tyrosine-protein phosphatase S	OID01284	Q13332	2.26	0.56	9.06	0.24993	0.46956
RARRES2	Retinoic acid receptor responder protein 2	OID00645	Q99969	3.56	1.33	9.50	0.01132	0.07726
REG1A	Lithostathine-1-alpha	OID01231	P05451	1.10	0.64	1.91	0.72906	0.84709
RETN	Resistin	OID00603	Q9HD89	1.50	0.81	2.78	0.19377	0.41073
SAA4	Serum amyloid A-4 protein	OID01269	P35542	1.11	0.65	1.89	0.70419	0.83939
SCGB3A2	Secretoglobin family 3A member 2	OID00636	Q96PL1	1.43	1.10	1.88	0.00820	0.06780
SELE	E-selectin	OID00596	P16581	1.26	0.70	2.27	0.43468	0.63042
SELL	L-selectin	OID01249	P14151	1.46	0.55	3.88	0.45153	0.63989
SELP	P-selectin	OID00574	P16109	1.39	1.01	1.91	0.04244	0.15708
SERPINA5	Plasma serine protease inhibitor	OID01229	P05154	0.70	0.33	1.51	0.36519	0.57397
SERPINA7	Thyroxine-binding globulin	OID01232	P05543	1.18	0.49	2.83	0.71651	0.83939
SHPS1	Tyrosine-protein phosphatase non-receptor type substrate 1	OID00628	P78324	3.75	1.57	8.93	0.00285	0.03369
SOD1	Superoxide dismutase [Cu-Zn]	OID01222	P00441	1.25	0.93	1.69	0.13632	0.32507
SPARCL1	SPARC-like protein 1	OID01287	Q14515	1.24	0.36	4.29	0.73095	0.84709
ST2	Interleukin-1 receptor-like 1	OID00634	Q01638	0.87	0.45	1.65	0.66382	0.83939
ST6GAL1	Beta-galactoside alpha-2,6-sialyltransferase 1	OID01252	P15907	1.25	0.89	1.75	0.20562	0.41458
TPA	Tissue-type plasminogen activator chain A	OID00635	P00750	1.04	0.70	1.55	0.84463	0.91073
TCN2	Transcobalamin-2	OID01259	P20062	2.68	1.35	5.32	0.00500	0.04772
TFF3	Trefoil factor 3	OID00573	Q07654	1.47	0.72	3.01	0.29304	0.50468
TGFBI	Transforming growth factor-beta-induced protein ig-h3	OID01291	Q15582	0.85	0.37	1.95	0.69884	0.83939
TGFBR3	Transforming growth factor beta receptor type 3	OID01279	Q03167	1.63	0.58	4.60	0.35241	0.57123
THBS4	Thrombospondin-4	OID01268	P35443	1.26	0.77	2.06	0.36459	0.57397
TIE1	Tyrosine-protein kinase receptor Tie-1	OID01270	P35590	0.66	0.16	2.79	0.57453	0.76671
TIE2	Angiopoietin-1 receptor	OID00754	Q02763	0.42	0.13	1.36	0.15019	0.34811
TIMD4	T-cell immunoglobulin and mucin domain-containing protein 4	OID01298	Q96H15	1.66	0.89	3.07	0.10824	0.27961
TIMP1	Metalloproteinase inhibitor 1	OID01224	P01033	1.41	0.94	2.13	0.09763	0.26607
TIMP4	Metalloproteinase inhibitor 4	OID00585	Q99727	1.27	0.76	2.13	0.36171	0.57397
TLT2	Trem-like transcript 2 protein	OID00588	Q5T2D2	1.93	0.95	3.91	0.06769	0.20472
TNC	Tenascin	OID01265	P24821	1.70	0.95	3.06	0.07338	0.21664
TNF	Tumor necrosis factor, membrane form	OID05554	P01375	1.67	0.99	2.81	0.05634	0.17915
TNFR1	Tumor necrosis factor receptor superfamily member 1A, membrane form	OID00649	P19438	3.04	1.22	7.58	0.01681	0.09308
TNFR2	Tumor necrosis factor receptor superfamily member 1b, membrane form	OID00567	P20333	1.17	0.52	2.65	0.70102	0.83939
TNFRSF10C	Tumor necrosis factor receptor superfamily member 10C	OID00594	O14798	1.29	0.60	2.77	0.52238	0.70792

Protein	Name	Olink ID	UniProt	aHR	95% lower CI	95% higher CI	p	FDR
TNFRSF12A	Tumor necrosis factor receptor superfamily member 12A	OID00810	Q9NP84	2.31	1.04	5.15	0.04003	0.15274
TNFRSF14	Tumor necrosis factor receptor superfamily member 14	OID00563	Q92956	2.13	1.13	4.02	0.01875	0.09957
TNFRSF21	Tumor necrosis factor receptor superfamily member 21	OID00818	O75509	2.78	0.62	12.57	0.18388	0.39313
TNFRSF4	Tumor necrosis factor receptor superfamily member 4	OID00819	P43489	1.98	1.05	3.70	0.03363	0.14137
TNFRSF9	Tumor necrosis factor receptor superfamily member 9	OID00753	Q07011	2.35	1.33	4.15	0.00328	0.03698
TNFSF13B	Tumor necrosis factor ligand superfamily member 13b, membrane form	OID00617	Q9Y275	3.33	1.85	6.02	0.00007	0.00595
TNFSF14	Tumor necrosis factor ligand superfamily member 14, membrane form	OID00787	O43557	1.64	1.10	2.45	0.01552	0.08952
TNXB	Tenascin-X	OID01260	P22105	1.74	0.52	5.84	0.36838	0.57458
TR	Transferrin receptor protein 1, serum form	OID00593	P02786	1.58	0.91	2.74	0.10594	0.27656
TRAP	Tartrate-resistant acid phosphatase type 5	OID00606	P13686	0.84	0.33	2.16	0.71672	0.83939
TWEAK	Tumor necrosis factor ligand superfamily member 12, membrane form	OID00789	O43508	0.68	0.27	1.75	0.42385	0.62282
UPAR	Urokinase plasminogen activator surface receptor	OID00620	Q03405	2.68	1.65	4.36	0.00007	0.00595
UPA	Urokinase-type plasminogen activator short chain A	OID00631	P00749	1.17	0.57	2.41	0.67155	0.83939
VASN	Vasorin	OID01295	Q6EMK4	1.08	0.27	4.29	0.90947	0.94063
VCAM1	Vascular cell adhesion protein 1	OID01257	P19320	3.03	1.29	7.10	0.01077	0.07726
VEGFA	Vascular endothelial growth factor A	OID00832	P15692	3.45	1.83	6.49	0.00013	0.00784
VEGFR2	Vascular endothelial growth factor receptor 2	OID00780	P35968	0.68	0.18	2.60	0.57503	0.76671
VWF	Von Willebrand antigen 2	OID00651	P04275	1.73	1.08	2.76	0.02156	0.10911

Models were corrected for 10-year ASCVD risk score and statin use. Estimates are provided per doubling in protein expression from Cox regression models.
Abbreviations: CI: confidence interval; FDR: False discovery rate corrected p value; Olink ID: protein ID in Olink database; UniProt: UniProt ID of protein.

Supplemental table 5. Association between proteomic markers and hard MACE.

Protein	Name	Olink ID	UniProt	aHR	95% lower CI	95% higher CI	p	FDR
ADA	Adenosine deaminase	OID00775	P00813	1.79	0.95	3.38	0.07059	0.20988
ALCAM	CD166 antigen	OID00572	Q13740	2.14	0.75	6.09	0.15346	0.32332
ANG	Angiogenin	OID01226	P03950	0.96	0.38	2.42	0.92998	0.95699
ANGPT1	Angiopoietin-1	OID00760	Q15389	1.79	1.10	2.92	0.01980	0.12920
ANGPT2	Angiopoietin-2	OID00822	O15123	2.98	1.22	7.30	0.01694	0.12361
AOC3	Membrane primary amine oxidase	OID01294	Q16853	0.85	0.23	3.16	0.81430	0.89035
APN	Aminopeptidase N	OID00611	P15144	0.36	0.08	1.54	0.16960	0.33772
APOM	Apolipoprotein M	OID01221	O95445	0.55	0.19	1.57	0.26443	0.44515
ARG1	Arginase-1	OID00815	P05089	0.98	0.57	1.68	0.92901	0.95699
AXL	Tyrosine-protein kinase receptor UFO	OID00612	P30530	1.38	0.40	4.82	0.61191	0.77425
AZU1	Azurocidin	OID00597	P20160	1.40	0.85	2.31	0.18880	0.36581
BLM HYDROLASE	Bleomycin hydrolase	OID00581	Q13867	1.35	0.64	2.88	0.43083	0.60026
C1QTNF1	Complement C1q tumor necrosis factor-related protein 1	OID01301	Q9BXJ1	0.88	0.41	1.86	0.73090	0.84079
C2	Complement C2a fragment	OID01233	P06681	2.25	0.60	8.46	0.22900	0.41153
CA1	Carbonic anhydrase 1	OID01223	P00915	1.08	0.67	1.75	0.73824	0.84370
CA3	Carbonic anhydrase 3	OID01235	P07451	1.29	0.62	2.68	0.49590	0.66477
CA4	Carbonic anhydrase 4	OID01261	P22748	1.59	0.76	3.30	0.21544	0.39872
CAIX	Carbonic anhydrase 9	OID00773	Q16790	1.47	0.63	3.42	0.37132	0.55811
CASP3	Caspase-3 subunit p12	OID00630	P42574	1.34	1.01	1.78	0.04092	0.17955
CASP8	Caspase-8 subunit p10	OID00827	Q14790	1.64	1.04	2.60	0.03457	0.16810
CCL14	C-C motif chemokine 14	OID01292	Q16627	2.04	0.81	5.15	0.13183	0.30273
CCL15	C-C motif chemokine 15	OID00629	Q16663	1.17	0.56	2.44	0.66743	0.80564
CCL16	C-C motif chemokine 16	OID00654	O15467	1.03	0.58	1.83	0.91225	0.95459
CCL17	C-C motif chemokine 17	OID00821	Q92583	1.33	0.93	1.89	0.12190	0.28792
CCL18	C-C motif chemokine 18	OID01276	P55774	1.33	0.73	2.41	0.34771	0.52902
CCL19	C-C motif chemokine 19	OID00794	Q99731	1.44	0.87	2.38	0.15172	0.32332
CCL20	C-C motif chemokine 20	OID00837	P78556	0.92	0.59	1.43	0.70125	0.82422
CCL23	C-C motif chemokine 23	OID00811	P55773	2.22	0.98	5.03	0.05645	0.20354
CCL24	C-C motif chemokine 24	OID00592	O00175	0.70	0.41	1.20	0.19420	0.37047
CCL3	C-C motif chemokine 3	OID00813	P10147	1.23	0.93	1.62	0.14062	0.31702
CCL4	C-C motif chemokine 4	OID00796	P13236	0.96	0.49	1.90	0.90587	0.95459
CCL5	C-C motif chemokine 5	OID01246	P13501	1.29	1.05	1.59	0.01696	0.12361
CD163	Scavenger receptor cysteine-rich type 1 protein M130	OID00577	Q86VB7	0.84	0.37	1.92	0.67742	0.80769
CD244	Natural killer cell receptor 2B4	OID00758	Q9BZW8	1.53	0.83	2.80	0.17022	0.33772
CD27	CD27 antigen	OID00800	P26842	3.56	1.32	9.59	0.01201	0.11732
CD4	T-cell surface glycoprotein CD4	OID00776	P01730	1.48	0.61	3.61	0.38524	0.56587
CD40	Tumor necrosis factor receptor superfamily member 5	OID00781	P25942	2.56	1.24	5.30	0.01110	0.11471
CD40L	CD40 ligand, membrane form	OID00756	P29965	1.41	1.06	1.87	0.01744	0.12361
CD46	Membrane cofactor protein	OID01251	P15529	1.36	0.99	1.85	0.05423	0.20354
CD5	T-cell surface glycoprotein CD5	OID00812	P06127	1.79	0.64	4.99	0.26394	0.44515
CD59	CD59 glycoprotein	OID01248	P13987	1.49	0.70	3.16	0.29788	0.48232
CD70	CD70 antigen	OID00808	P32970	3.16	1.55	6.47	0.00160	0.04145
CD83	CD83 antigen	OID00841	Q01151	2.63	1.20	5.73	0.01519	0.11775
CD8A	T-cell surface glycoprotein CD8 alpha chain	OID00772	P01732	1.19	0.66	2.15	0.56941	0.73549
CD93	Complement component C1q receptor	OID00639	Q9NPY3	2.24	0.65	7.78	0.20393	0.38314
CDH1	Cadherin-1	OID01245	P12830	1.89	0.78	4.61	0.15880	0.32332
CDH5	Cadherin-5	OID00587	P33151	0.84	0.24	2.94	0.78334	0.87904
CES1	Liver carboxylesterase 1	OID01263	P23141	1.05	0.61	1.83	0.85196	0.91466
CFHR5	Complement factor H-related protein 5	OID01302	Q9BXR6	1.60	0.62	4.12	0.32989	0.51455
CHI3L1	Chitinase-3-like protein 1	OID00633	P36222	1.17	0.81	1.70	0.40568	0.58494
CHIT1	Chitotriosidase-1	OID00605	Q13231	0.84	0.63	1.13	0.25728	0.44003
CHL1	Processed neural cell adhesion molecule L1-like protein	OID01216	O00533	1.63	0.36	7.44	0.52745	0.69257
CNDP1	Carnosine dipeptidase 1	OID01299	Q96KN2	0.93	0.51	1.71	0.81496	0.89035

Protein	Name	Olink ID	UniProt	aHR	95% lower CI	95% higher CI	p	FDR
CNTN1	Contactin-1	OID00586	Q12860	0.30	0.07	1.25	0.09830	0.25394
COL18A1	Collagen alpha-1(XVIII) chain	OID01271	P39060	2.13	0.65	6.95	0.21105	0.39353
COL1A1	Collagen alpha-1(I) chain	OID00641	P02452	2.58	0.91	7.34	0.07519	0.21683
COMP	Cartilage oligomeric matrix protein	OID01274	P49747	0.85	0.35	2.04	0.71517	0.83241
CPA1	Carboxypeptidase A1	OID00624	P15085	1.42	0.85	2.37	0.18467	0.36195
CPB1	Carboxypeptidase B1	OID00632	P15086	1.51	0.90	2.52	0.11487	0.27863
CR2	Complement receptor type 2	OID01258	P20023	0.54	0.24	1.20	0.13033	0.30207
CRTAC1	Cartilage acidic protein 1	OID01304	Q9NQ79	0.55	0.21	1.48	0.23921	0.42228
CRTAM	Cytotoxic and regulatory T-cell molecule	OID00766	O95727	1.33	0.70	2.51	0.38023	0.56466
CSF1	Processed macrophage colony-stimulating factor 1	OID00843	P09603	19.92	3.77	105.39	0.00043	0.02688
CST3	Cystatin-C	OID01225	P01034	3.00	1.11	8.10	0.03032	0.16507
CSTB	Cystatin-B	OID00575	P04080	1.81	1.13	2.88	0.01348	0.11732
CTSD	Cathepsin D heavy chain	OID00622	P07339	1.10	0.48	2.49	0.82490	0.89334
CTSZ	Cathepsin Z	OID00643	Q9UBR2	2.74	0.84	8.95	0.09486	0.25027
CX3CL1	Processed fractalkine	OID00806	P78423	2.93	0.91	9.39	0.07109	0.20988
CXCL1	Growth-regulated alpha protein	OID00786	P09341	1.38	0.94	2.04	0.10128	0.25505
CXCL10	C-X-C motif chemokine 10	OID00807	P02778	1.28	0.83	1.99	0.26745	0.44515
CXCL11	C-X-C motif chemokine 11	OID00767	O14625	1.20	0.84	1.71	0.32611	0.51187
CXCL12	Stromal cell-derived factor 1	OID00824	P48061	2.70	0.90	8.14	0.07760	0.22119
CXCL13	C-X-C motif chemokine 13	OID00830	O43927	1.65	1.16	2.34	0.00555	0.07651
CXCL16	C-X-C motif chemokine 16	OID00601	Q9H2A7	6.00	1.54	23.31	0.00970	0.11441
CXCL5	C-X-C motif chemokine 5	OID00801	P42830	1.36	0.99	1.88	0.05882	0.20354
CXCL9	C-X-C motif chemokine 9	OID00771	Q07325	1.22	0.75	1.96	0.42235	0.59177
DCN	Decorin	OID00817	P07585	0.96	0.38	2.42	0.92561	0.95699
DLK1	Protein delta homolog 1	OID00598	P80370	1.49	0.77	2.89	0.23446	0.41831
DPP4	Dipeptidyl peptidase 4 membrane form	OID01266	P27487	0.36	0.12	1.05	0.06067	0.20354
EFEMP1	EGF-containing fibulin-like extracellular matrix protein 1	OID01281	Q12805	3.57	1.58	8.04	0.00216	0.04145
EGF	Pro-epidermal growth factor	OID00759	P01133	1.41	1.03	1.93	0.03324	0.16810
EGFR	Epidermal growth factor receptor	OID00637	P00533	0.41	0.05	3.57	0.41718	0.59120
ENG	Endoglin	OID01254	P17813	1.28	0.45	3.67	0.64121	0.79909
EPCAM	Epithelial cell adhesion molecule	OID00610	P16422	0.96	0.59	1.56	0.88081	0.94156
EPHB4	Ephrin type-B receptor 4	OID00569	P54760	3.48	0.81	15.01	0.09444	0.25027
F11	Coagulation factor XIa heavy chain	OID01227	P03951	0.98	0.29	3.32	0.97478	0.97873
F7	Coagulation factor VII	OID01239	P08709	3.68	0.90	14.94	0.06887	0.20988
FABP4	Fatty acid-binding protein, adipocyte	OID00589	P15090	2.55	1.48	4.40	0.00075	0.02866
FAS	Tumor necrosis factor receptor superfamily member 6	OID00615	P25445	1.92	0.92	3.98	0.08099	0.22824
FASLG	Tumor necrosis factor ligand superfamily member 6, membrane form	OID00792	P48023	0.95	0.35	2.62	0.92586	0.95699
FCGR2A	Low affinity immunoglobulin gamma Fc region receptor II-a	OID01244	P12318	0.83	0.43	1.62	0.59443	0.75989
FCGR3B	Low affinity immunoglobulin gamma Fc region receptor III-B	OID01219	O75015	1.47	0.61	3.51	0.39082	0.57014
FCN2	Ficolin-2	OID01290	Q15485	1.88	0.83	4.24	0.13005	0.30207
FETUB	Fetuin-B	OID01305	Q9UGM5	1.14	0.47	2.79	0.77330	0.87172
FGF2	Fibroblast growth factor 2	OID00770	P09038	1.62	1.08	2.45	0.02096	0.12994
GAL1	Galectin-1	OID00798	P09382	2.70	0.50	14.66	0.25053	0.43448
GAL3	Galectin-3	OID00578	P17931	1.80	0.64	5.07	0.26926	0.44518
GAL4	Galectin-4	OID00626	P56470	1.27	0.64	2.54	0.49984	0.66646
GAL9	Galectin-9	OID00779	O00182	5.78	2.15	15.53	0.00050	0.02688
GAS6	Growth arrest-specific protein 6	OID01286	Q14393	3.41	1.08	10.77	0.03628	0.16977
GDF15	Growth/differentiation factor 15	OID00595	Q99988	1.78	0.93	3.43	0.08384	0.23361
GNLY	Granulysin	OID01262	P22749	0.92	0.47	1.77	0.79509	0.88058
GP1BA	Platelet glycoprotein Ib alpha chain	OID01234	P07359	1.44	1.08	1.92	0.01292	0.11732

Protein	Name	Olink ID	UniProt	aHR	95% lower CI	95% higher CI	p	FDR
GP6	Platelet glycoprotein VI	OID05026	Q9HCN6	1.63	1.01	2.63	0.04565	0.18560
GRN	Paragranulin	OID00579	P28799	6.38	2.00	20.42	0.00177	0.04145
GZMA	Granzyme A	OID00804	P12544	1.29	0.76	2.21	0.34508	0.52902
GZMB	Granzyme B	OID00840	P10144	1.09	0.73	1.63	0.66920	0.80564
GZMH	Granzyme H	OID00783	P20718	1.09	0.74	1.61	0.66524	0.80564
HGF	Hepatocyte growth factor alpha chain	OID00803	P14210	2.52	1.07	5.95	0.03441	0.16810
HO1	Heme oxygenase 1	OID00805	P09601	0.68	0.25	1.85	0.45261	0.62015
ICAM2	Intercellular adhesion molecule 2	OID00646	P13598	0.97	0.30	3.15	0.95566	0.96737
ICAM1	Intercellular adhesion molecule 1	OID01230	P05362	2.06	0.69	6.20	0.19768	0.37423
ICAM3	Intercellular adhesion molecule 3	OID01267	P32942	1.26	0.45	3.54	0.66665	0.80564
ICOSLG	Ig-like domain-containing protein	OID00828	O75144	0.18	0.03	1.14	0.06854	0.20988
IFNG	Interferon gamma	OID05552	P01579	1.37	0.98	1.91	0.06650	0.20988
IGFBP1	Insulin-like growth factor-binding protein 1	OID00604	P08833	0.95	0.65	1.40	0.79891	0.88058
IGFBP2	Insulin-like growth factor-binding protein 2	OID00650	P18065	1.54	0.89	2.66	0.12174	0.28792
IGFBP7	Insulin-like growth factor-binding protein 7	OID00638	Q16270	1.50	0.44	5.12	0.52008	0.68973
IGFBP3	Insulin-like growth factor-binding protein 3	OID01255	P17936	1.32	0.52	3.32	0.55862	0.72533
IGFBP6	Insulin-like growth factor-binding protein 6	OID01264	P24592	2.21	0.67	7.29	0.19406	0.37047
IGLC2	Ig lambda-2 chain C regions	OID01240	P0DOY2	1.92	0.95	3.91	0.07035	0.20988
IL17RA	Interleukin-17 receptor A	OID00566	Q96F46	0.98	0.47	2.06	0.95396	0.96737
IL18BP	Interleukin-18-binding protein	OID00640	O95998	2.55	0.91	7.13	0.07444	0.21683
IL1RT1	Interleukin-1 receptor type 1, membrane form	OID00613	P14778	1.09	0.24	5.02	0.91104	0.95459
IL1RT2	Interleukin-1 receptor type 2, membrane form	OID00627	P27930	0.75	0.21	2.65	0.65228	0.80480
IL6RA	Interleukin-6 receptor subunit alpha	OID00602	P08887	4.15	1.32	13.05	0.01473	0.11775
IL10	Interleukin-10	OID00809	P22301	1.09	0.56	2.11	0.79890	0.88058
IL12	Interleukin-12 subunit alpha and beta	OID00842	P29459, P29460	1.39	0.77	2.49	0.27474	0.44825
IL12RB1	Interleukin-12 receptor subunit beta-1	OID00835	P42701	1.59	0.65	3.89	0.31375	0.49879
IL15	Interleukin-15	OID05551	P40933	3.40	0.98	11.85	0.05471	0.20354
IL18	Interleukin-18	OID00782	Q14116	1.26	0.61	2.60	0.53703	0.70097
IL2RA	Interleukin-2 receptor subunit alpha	OID00570	P01589	2.18	0.97	4.91	0.06036	0.20354
IL6	Interleukin-6	OID00763	P05231	1.93	1.36	2.74	0.00024	0.02688
IL7	Interleukin-7	OID00761	P13232	1.50	1.00	2.24	0.04988	0.19634
IL7R	Interleukin-7 receptor subunit alpha	OID01253	P16871	0.65	0.23	1.84	0.42029	0.59177
IL8	Interleukin-8	OID00752	P10145	1.56	1.01	2.41	0.04298	0.18054
ITGB2	Integrin beta-2	OID00565	P05107	0.77	0.24	2.47	0.65648	0.80564
JAMA	Junctional adhesion molecule A	OID00625	Q9Y624	1.40	1.01	1.94	0.04078	0.17955
KIR3DL1	Killer cell immunoglobulin-like receptor 3DL1	OID05550	P43629	1.76	1.14	2.71	0.01061	0.11441
KIT	Mast/stem cell growth factor receptor Kit	OID01241	P10721	1.35	0.39	4.61	0.63424	0.79440
KLK6	Kallikrein-6	OID00647	Q92876	1.38	0.42	4.58	0.59877	0.76151
KLRD1	Natural killer cells antigen CD94	OID00839	Q13241	1.02	0.54	1.95	0.94000	0.96068
LAG3	Secreted lymphocyte activation gene 3 protein	OID05553	P18627	1.45	0.74	2.83	0.27319	0.44825
LAMP3	Lysosome-associated membrane glycoprotein 3	OID00826	Q9UQV4	2.06	1.19	3.57	0.01027	0.11441
LAP TGFB1	Transforming growth factor beta-1 proprotein	OID00785	P01137	1.70	1.00	2.88	0.04955	0.19634
LDL RECEPTOR	Low-density lipoprotein receptor	OID00564	P01130	1.14	0.52	2.48	0.74427	0.84669

Protein	Name	Olink ID	UniProt	aHR	95% lower CI	95% higher CI	p	FDR
LILRB1	Leukocyte immunoglobulin-like receptor subfamily B member 1	OID01297	Q8NHL6	1.46	0.59	3.60	0.41358	0.59120
LILRB2	Leukocyte immunoglobulin-like receptor subfamily B member 2	OID01296	Q8N423	1.46	0.76	2.80	0.25242	0.43473
LILRB5	Leukocyte immunoglobulin-like receptor subfamily B member 5	OID01220	O75023	1.41	0.68	2.93	0.35306	0.53389
LTBR	Tumor necrosis factor receptor superfamily member 3	OID00583	P36941	2.61	0.71	9.63	0.14888	0.32107
LYVE1	Lymphatic vessel endothelial hyaluronic acid receptor 1	OID01307	Q9Y5Y7	1.07	0.36	3.23	0.90100	0.95459
MB	Myoglobin	OID00616	P02144	1.47	0.79	2.77	0.22700	0.41153
MCP1	C-C motif chemokine 2	OID00576	P13500	3.19	1.21	8.46	0.01942	0.12920
MCP2	C-C motif chemokine 8	OID00795	P80075	2.37	1.37	4.09	0.00198	0.04145
MCP3	C-C motif chemokine 7	OID00755	P80098	2.19	1.32	3.66	0.00262	0.04637
MCP4	C-C motif chemokine 13, medium chain	OID00768	Q99616	1.70	1.05	2.77	0.03241	0.16744
MEGF9	Multiple epidermal growth factor-like domains protein 9	OID01303	Q9H1U4	1.15	0.29	4.47	0.84535	0.91151
MEPE	Matrix extracellular phosphoglycoprotein	OID00132	Q9NQ76	1.52	0.49	4.76	0.47195	0.63958
MET	Hepatocyte growth factor receptor	OID01238	P08581	6.28	0.88	44.50	0.06612	0.20988
MFAP5	Microfibrillar-associated protein 5	OID01285	Q13361	1.95	0.44	8.62	0.37647	0.56244
MMP2	72 kDa type IV collagenase	OID00614	P08253	1.37	0.35	5.39	0.65106	0.80480
MMP3	Stromelysin-1	OID00644	P08254	0.75	0.35	1.57	0.44109	0.60773
MMP9	67 kDa matrix metalloproteinase-9	OID00568	P14780	1.23	0.68	2.23	0.48590	0.65491
MMP12	Macrophage metalloelastase	OID00829	P39900	1.08	0.59	1.98	0.79778	0.88058
MMP7	Matrilysin	OID00814	P09237	1.90	0.89	4.06	0.09635	0.25153
MPO	Myeloperoxidase heavy chain	OID00600	P05164	0.67	0.22	1.99	0.46786	0.63752
MUC16	Mucin-16	OID05549	Q8WXI7	1.12	0.61	2.05	0.70731	0.82741
NCAM1	Neural cell adhesion molecule 1	OID01247	P13591	1.08	0.27	4.39	0.91188	0.95459
NCR1	Natural cytotoxicity triggering receptor 1	OID00816	O76036	3.41	1.27	9.16	0.01477	0.11775
NID1	Nidogen-1	OID01250	P14543	1.46	1.01	2.11	0.04224	0.18054
NOS3	Nitric oxide synthase, endothelial	OID00777	P29474	2.18	1.07	4.41	0.03128	0.16507
NOTCH 3	Neurogenic locus notch homolog protein 3	OID00584	Q9UM47	1.38	0.44	4.38	0.58363	0.74995
NOTCH1	Neurogenic locus notch homolog protein 1	OID01273	P46531	4.20	0.61	28.75	0.14343	0.31702
NT-PROBNP	NT-proBNP	OID00131	NA	1.97	1.24	3.13	0.00409	0.06760
OPG	Tumor necrosis factor receptor superfamily member 11B	OID00571	O00300	2.53	0.94	6.79	0.06619	0.20988
OPN	Osteopontin	OID00621	P10451	2.21	1.33	3.65	0.00208	0.04145
OSMR	Oncostatin-M-specific receptor subunit beta	OID01300	Q99650	8.99	1.25	64.50	0.02900	0.16346
PAI	Plasminogen activator inhibitor 1	OID00591	P05121	1.61	1.00	2.59	0.05191	0.20114
PAM	Peptidyl-alpha-hydroxyglycine alpha-amidating lyase	OID01256	P19021	2.33	0.56	9.67	0.24558	0.42890
PCSK9	Proprotein convertase subtilisin/kexin type 9	OID00619	Q8NBP7	3.75	0.95	14.78	0.05895	0.20354
PDL1	Programmed cell death 1 ligand 1	OID00799	Q9NZQ7	2.39	1.13	5.04	0.02206	0.13345
PDL2	Programmed cell death 1 ligand 2	OID00831	Q9BQ51	1.26	0.39	4.02	0.69841	0.82422
PDCD1	Programmed cell death protein 1	OID00791	Q15116	2.10	1.07	4.13	0.03062	0.16507
PDGF SUBUNIT A	Platelet-derived growth factor subunit A	OID00648	P04085	1.62	1.07	2.45	0.02379	0.14049
PDGF SUBUNIT B	Platelet-derived growth factor subunit B	OID00790	P01127	2.39	1.11	5.13	0.02529	0.14586
PECAM1	Platelet endothelial cell adhesion molecule	OID00652	P16284	1.56	1.02	2.38	0.04127	0.17955
PGF	Placenta growth factor	OID00762	P49763	2.06	0.46	9.26	0.34615	0.52902

Protein	Name	Olink ID	UniProt	aHR	95% lower CI	95% higher CI	p	FDR
PGLYRP1	Peptidoglycan recognition protein 1	OID00623	O75594	1.85	0.78	4.40	0.16274	0.32812
PI3	Elafin	OID00609	P19957	1.03	0.49	2.17	0.94131	0.96068
PLC	Basement membrane-specific heparan sulfate proteoglycan core protein	OID00582	P98160	2.76	0.61	12.36	0.18535	0.36195
PLTP	Phospholipid transfer protein	OID01275	P55058	1.99	0.65	6.06	0.22800	0.41153
PLXNB2	Plexin-B2	OID01218	O15031	1.66	0.52	5.33	0.39537	0.57340
PON3	Serum paraoxonase/lactonase 3	OID00642	Q15166	0.48	0.28	0.83	0.00864	0.10712
PROC	Vitamin K-dependent protein C heavy chain	OID01228	P04070	0.81	0.25	2.64	0.73230	0.84079
PRSS2	Trypsin-2	OID01236	P07478	1.86	0.96	3.59	0.06447	0.20988
PRTN3	Myeloblastin	OID00618	P24158	1.40	0.62	3.18	0.41623	0.59120
PSPD	Pulmonary surfactant-associated protein D	OID00608	P35247	1.49	0.77	2.90	0.24009	0.42228
PTPRS	Receptor-type tyrosine-protein phosphatase S	OID01284	Q13332	4.57	1.04	20.03	0.04368	0.18054
RARRES2	Retinoic acid receptor responder protein 2	OID00645	Q99969	8.96	2.59	30.93	0.00053	0.02688
REG1A	Lithostathine-1-alpha	OID01231	P05451	1.38	0.75	2.54	0.30300	0.48480
RETN	Resistin	OID00603	Q9HD89	2.10	0.97	4.55	0.06073	0.20354
SAA4	Serum amyloid A-4 protein	OID01269	P35542	0.88	0.44	1.75	0.71829	0.83241
SCGB3A2	Secretoglobin family 3A member 2	OID00636	Q96PL1	1.32	0.90	1.93	0.15905	0.32332
SELE	E-selectin	OID00596	P16581	1.39	0.66	2.93	0.38561	0.56587
SELL	L-selectin	OID01249	P14151	2.04	0.58	7.12	0.26570	0.44515
SELP	P-selectin	OID00574	P16109	1.61	1.08	2.39	0.01896	0.12920
SERPINA5	Plasma serine protease inhibitor	OID01229	P05154	1.24	0.42	3.69	0.69548	0.82422
SERPINA7	Thyroxine-binding globulin	OID01232	P05543	1.02	0.34	3.07	0.96512	0.97296
SHPS1	Tyrosine-protein phosphatase non-receptor type substrate 1	OID00628	P78324	2.56	0.82	8.04	0.10668	0.26457
SOD1	Superoxide dismutase [Cu-Zn]	OID01222	P00441	1.41	0.99	2.02	0.05797	0.20354
SPARCL1	SPARC-like protein 1	OID01287	Q14515	3.27	0.64	16.79	0.15645	0.32332
ST2	Interleukin-1 receptor-like 1	OID00634	Q01638	0.91	0.39	2.09	0.81994	0.89186
ST6GAL1	Beta-galactoside alpha-2,6-sialyltransferase 1	OID01252	P15907	1.38	0.94	2.04	0.10155	0.25505
TPA	Tissue-type plasminogen activator chain A	OID00635	P00750	1.37	0.90	2.08	0.13950	0.31702
TCN2	Transcobalamin-2	OID01259	P20062	3.44	1.44	8.21	0.00536	0.07651
TFF3	Trefoil factor 3	OID00573	Q07654	1.70	0.72	4.02	0.22701	0.41153
TGFBI	Transforming growth factor-beta-induced protein ig-h3	OID01291	Q15582	0.80	0.28	2.26	0.67463	0.80769
TGFBR3	Transforming growth factor beta receptor type 3	OID01279	Q03167	2.95	0.86	10.16	0.08588	0.23664
THBS4	Thrombospondin-4	OID01268	P35443	1.36	0.72	2.54	0.34193	0.52902
TIE1	Tyrosine-protein kinase receptor Tie-1	OID01270	P35590	0.28	0.05	1.64	0.15767	0.32332
TIE2	Angiopoietin-1 receptor	OID00754	Q02763	0.27	0.06	1.25	0.09401	0.25027
TIMD4	T-cell immunoglobulin and mucin domain-containing protein 4	OID01298	Q96H15	1.51	0.67	3.39	0.31787	0.50211
TIMP1	Metalloproteinase inhibitor 1	OID01224	P01033	1.50	0.91	2.45	0.10884	0.26725
TIMP4	Metalloproteinase inhibitor 4	OID00585	Q99727	1.53	0.86	2.73	0.14415	0.31702
TLT2	Trem-like transcript 2 protein	OID00588	Q5T2D2	2.49	1.06	5.84	0.03624	0.16977
TNC	Tenascin	OID01265	P24821	1.73	0.83	3.64	0.14586	0.31730
TNF	Tumor necrosis factor, membrane form	OID05554	P01375	1.73	0.92	3.25	0.08705	0.23723
TNFR1	Tumor necrosis factor receptor superfamily member 1A, membrane form	OID00649	P19438	5.12	1.65	15.84	0.00465	0.07200
TNFR2	Tumor necrosis factor receptor superfamily member 1b, membrane form	OID00567	P20333	3.10	1.27	7.58	0.01330	0.11732
TNFRSF10C	Tumor necrosis factor receptor superfamily member 10C	OID00594	O14798	1.37	0.51	3.68	0.52780	0.69257

Protein	Name	Olink ID	UniProt	aHR	95% lower CI	95% higher CI	p	FDR
TNFRSF12A	Tumor necrosis factor receptor superfamily member 12A	OID00810	Q9NP84	2.14	0.74	6.17	0.15862	0.32332
TNFRSF14	Tumor necrosis factor receptor superfamily member 14	OID00563	Q92956	2.66	1.30	5.42	0.00724	0.09455
TNFRSF21	Tumor necrosis factor receptor superfamily member 21	OID00818	O75509	7.94	1.09	57.96	0.04105	0.17955
TNFRSF4	Tumor necrosis factor receptor superfamily member 4	OID00819	P43489	1.94	0.85	4.42	0.11572	0.27863
TNFRSF9	Tumor necrosis factor receptor superfamily member 9	OID00753	Q07011	2.11	0.97	4.56	0.05824	0.20354
TNFSF13B	Tumor necrosis factor ligand superfamily member 13b, membrane form	OID00617	Q9Y275	3.55	1.58	7.98	0.00217	0.04145
TNFSF14	Tumor necrosis factor ligand superfamily member 14, membrane form	OID00787	O43557	1.80	1.10	2.97	0.02043	0.12993
TNXB	Tenascin-X	OID01260	P22105	0.99	0.17	5.58	0.98699	0.98699
TR	Transferrin receptor protein 1, serum form	OID00593	P02786	1.46	0.72	2.97	0.29950	0.48232
TRAP	Tartrate-resistant acid phosphatase type 5	OID00606	P13686	2.34	0.75	7.30	0.14445	0.31702
TWEAK	Tumor necrosis factor ligand superfamily member 12, membrane form	OID00789	O43508	1.36	0.39	4.73	0.62749	0.78994
UPAR	Urokinase plasminogen activator surface receptor	OID00620	Q03405	2.81	1.53	5.13	0.00081	0.02866
UPA	Urokinase-type plasminogen activator short chain A	OID00631	P00749	0.82	0.25	2.72	0.74851	0.84763
VASN	Vasorin	OID01295	Q6EMK4	4.31	0.75	24.77	0.10181	0.25505
VCAM1	Vascular cell adhesion protein 1	OID01257	P19320	2.79	0.93	8.40	0.06730	0.20988
VEGFA	Vascular endothelial growth factor A	OID00832	P15692	4.12	1.85	9.18	0.00054	0.02688
VEGFR2	Vascular endothelial growth factor receptor 2	OID00780	P35968	2.15	0.31	14.86	0.43599	0.60405
VWF	Von Willebrand antigen 2	OID00651	P04275	2.04	1.16	3.61	0.01372	0.11732

Models were corrected for 10-year ASCVD risk score and statin use. Hard MACE was defined as: cardiovascular death, myocardial infarction or stroke. Estimates are provided per doubling in protein expression from Cox regression models.

Abbreviations: CI: confidence interval; FDR: False discovery rate corrected p value; Olink ID: protein ID in Olink database; UniProt: UniProt ID of protein.

Supplemental table 6. List of proteins in enriched functions and pathways in order of signal strength.

Term ID	Term description	Proteins
**Biological Process (Gene Ontology) enrichment**		
GO:0002690	Positive regulation of leukocyte chemotaxis	CXCL13, IL8, CSF1, MCP3, GAL9, IL6, RARRES2
GO:0002687	Positive regulation of leukocyte migration	CXCL13, IL8, CSF1, MCP3, MCP2, GAL9, IL6, RARRES2
GO:0097529	Myeloid leukocyte migration	CXCL13, IL8, CSF1, MCP3, MCP2, IL6, SHPS1
GO:0050920	Regulation of chemotaxis	CXCL13, IL8, ANGPT2, CSF1, MCP3, GAL9, IL6, RARRES2
GO:0030335	Positive regulation of cell migration	GRN, HGF, CXCL13, NOS3, IL8, CSF1, VEGF, MCP3, MCP2, GAL9, IL6, RARRES2
GO:0022409	Positive regulation of cell-cell adhesion	CXCL13, IL7R, UPAR, TNFSF13B, CD83, GAL9, IL6, CD70, SHPS1
GO:0006959	Humoral immune response	CXCL13, IL8, PDCD1, NPPB, CD83, MCP2, IL6, RARRES2
GO:0032733	Positive regulation of interleukin-10 production	HGF, CD83, GAL9, IL6
GO:0045765	Regulation of angiogenesis	GRN, HGF, CXCL13, NOS3, IL8, ANGPT2, NPPB, IL6
GO:0019221	Cytokine-mediated signaling pathway	CXCL13, IL7R, IL8, CSF1, TNFSF13B, MCP3, MCP2, IL6, CD70
**Molecular Function (Gene Ontology) enrichment**		
GO:0005125	Cytokine activity	GRN, CXCL13, IL8, CSF1, TNFSF13B, MCP3, MCP2, OPN, IL6, CD70
GO:0048018	Receptor ligand activity	GRN, HGF, CXCL13, IL8, CSF1, TNFSF13B, NPPB, MCP3, EFEMP1, MCP2, OPN, IL6, CD70
GO:0005126	Cytokine receptor binding	CXCL13, IL8, CSF1, TNFSF13B, MCP3, MCP2, IL6, CD70
GO:0008009	Chemokine activity	CXCL13, IL8, MCP3, MCP2
GO:0005102	Signaling receptor binding	GRN, HGF, FABP4, CXCL13, IL8, ANGPT2, CSF1, UPAR, TNFSF13B, NPPB, MCP3, EFEMP1, MCP2, OPN, IL6, CD70, RARRES2
GO:0008083	Growth factor activity	GRN, HGF, CSF1, EFEMP1, IL6
GO:0008201	Heparin binding	CXCL13, IL8, VEGF, MCP3, MCP2
GO:0048020	CCR chemokine receptor binding	CXCL13, MCP3, MCP2
GO:0098772	Molecular function regulator activity	GRN, HGF, CXCL13, IL8, CSF1, TNFSF13B, NPPB, MCP3, EFEMP1, MCP2, OPN, IL6, CD70, SHPS1
GO:0005515	Protein binding	GRN, HGF, FABP4, PON3, CXCL13, NOS3, IL8, ANGPT2, CSF1, UPAR, VEGF, TNFSF13B, NPPB, MCP3, EFEMP1, MCP2, OPN, GAL9, IL6, CD70, RARRES2, TNFRSF9, SHPS1
**Reactome Pathways enrichment**		
HSA-168256	Immune System	GRN, HGF, NOS3, IL7R, IL8, CSF1, PDCD1, UPAR, TNFSF13B, GAL9, IL6, CD70, TNFRSF9, SHPS1
HSA-1280215	Cytokine Signaling in Immune system	HGF, IL7R, IL8, CSF1, TNFSF13B, GAL9, IL6, CD70, TNFRSF9
HSA-5669034	TNFs bind their physiological receptors	TNFSF13B, CD70, TNFRSF9
HSA-449147	Signaling by Interleukins	HGF, IL7R, IL8, CSF1, GAL9, IL6
HSA-6783783	Interleukin-10 signaling	IL8, CSF1, IL6
HSA-380108	Chemokine receptors bind chemokines	CXCL13, IL8, MCP3

Abbreviations: ANGPT2, Angiopoietin-2; CD27, CD27 antigen; CD70, CD70 antigen; CD83, CD83 antigen; CSF1, Processed macrophage colony-stimulating factor 1; CSTB, Cystatin-B; CXCL12, C-X-C motif chemokine 12; CXCL13, C-X-C motif chemokine 13; EFEMP1, EGF-containing fibulin-like extracellular matrix protein 1; FABP4, Fatty acid-binding protein, adipocyte; FDR, False discover rate; GAL9, Galectin-9; GDF15, Growth/differentiation factor 15; GRN, Paragranulin; IL6, Interleukin-6; IL7R, Interleukin-7 receptor subunit alpha; IL8, Interleukin-8; LAMP3, Lysosome-associated membrane glycoprotein 3; LAP TGFB1, Transforming growth factor beta-1 proprotein; LDL-C, low-density lipoprotein cholesterol; MCP2, C-C motif chemokine 8; MCP3, C-C motif chemokine 7; MCP4, C-C motif chemokine 13, medium chain; NOS3, Nitric oxide synthase, endothelial; NT-PROBNP, NT-proBNP; OPN, Osteopontin; PDCD1, Programmed cell death protein 1; PON3, Serum paraoxonase/lactonase 3; RARRES2, Retinoic acid receptor responder protein 2; SCGB3A2, Secretoglobin family 3A member 2; SHPS1, Tyrosine-protein phosphatase non-receptor type substrate 1; TCN2, Transcobalamin-2; TNFRSF9, Tumor necrosis factor receptor superfamily member 9; TNFSF13B, Tumor necrosis factor ligand superfamily member 13b, membrane form; UPAR, Urokinase plasminogen activator surface receptor; VEGFA, Vascular endothelial growth factor A

Supplemental table 7. Names of participating sites and their locations.

Site Name	Location
Alabama	Birmingham, Alabama, United States
University of Southern California	Los Angeles, California, United States
UCLA CARE Center	Los Angeles, California, United States
UCSD Antiviral Research Center	San Diego, California, United States
Ucsf Hiv/Aids	San Francisco, California, United States
Harbor-UCLA	Torrance, California, United States
University of Colorado Hospital	Aurora, Colorado, United States
Northwestern University	Chicago, Illinois, United States
Rush University	Chicago, Illinois, United States
Johns Hopkins University	Baltimore, Maryland, United States
Massachusetts General Hospital	Boston, Massachusetts, United States
Brigham and Women's Hospital Therapeutics Clinical Research Site	Boston, Massachusetts, United States
Washington University Therapeutics (WT)	St Louis, Missouri, United States
New Jersey Medical School Clinical Research Center	Newark, New Jersey, United States
Mount Sinai Beth Israel	New York, New York, United States
Weill Cornell Chelsea	New York, New York, United States
Mount Sinai Downtown	New York, New York, United States
Mount Sinai West Samuels	New York, New York, United States
Mount Sinai St. Luke's Morningside	New York, New York, United States
Columbia P&S	New York, New York, United States
Weill Cornell Uptown	New York, New York, United States
University of Rochester Adult HIV Therapeutic Strategies Network	Rochester, New York, United States
Chapel Hill	Chapel Hill, North Carolina, United States
Greensboro	Greensboro, North Carolina, United States
Cincinnati Clinical Research Site	Cincinnati, Ohio, United States
Case Clinical Research Site	Cleveland, Ohio, United States
Ohio State University	Columbus, Ohio, United States
Penn Therapeutics	Philadelphia, Pennsylvania, United States
University of Pittsburgh	Pittsburgh, Pennsylvania, United States
The Miriam Hospital Clinical Research Site	Providence, Rhode Island, United States
Vanderbilt Therapeutics (VT)	Nashville, Tennessee, United States
Houston AIDS Research Team	Houston, Texas, United States
University of Washington AIDS	Seattle, Washington, United States

SUPPLEMENTAL FIGURES

Supplemental figure 1. Proteins excluded from analysis due to quality assurance.

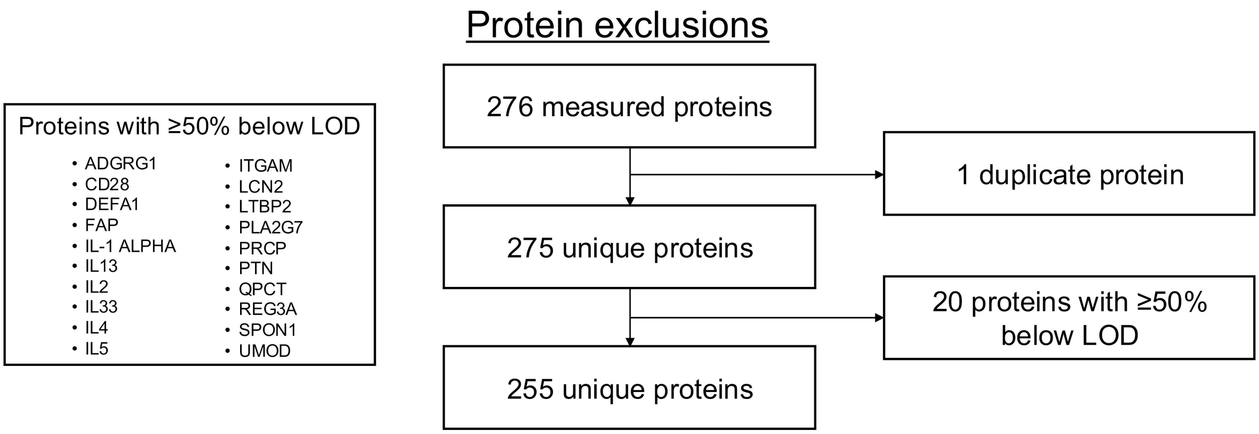

Following quality assurance and quality control measures described in the methods section, 255 proteins were used in proteomic analyses.
Abbreviations: ADGRG1, Adhesion G-protein coupled receptor G1; CD28, T-cell-specific surface glycoprotein CD28; DEFA1B, Neutrophil defensin 1; FAP, Antiplasmin-cleaving enzyme FAP, soluble form; IL1A, Interleukin-1 alpha; IL13, Interleukin-13; IL2, Interleukin-2; IL33, Interleukin-33; IL4, Interleukin-4; IL5, Interleukin-5; ITGAM, Integrin alpha-M; LCN2, Neutrophil gelatinase-associated lipocalin; LOD, limit of detection; LTBP2, Latent-transforming growth factor beta-binding protein 2; PLA2G7, Platelet-activating factor acetylhydrolase; PRCP, Lysosomal Pro-X carboxypeptidase; PTN, Pleiotrophin; QPCT, Glutaminyl-peptide cyclotransferase; REG3A, Regenerating islet-derived protein 3-alpha 16.5 kDa form; SPON1, Spondin-1; UMOD, Uromodulin, secreted form.

Supplemental figure 2. Association between proteomic markers and MACE or hard MACE – adjusting models using statin randomization.

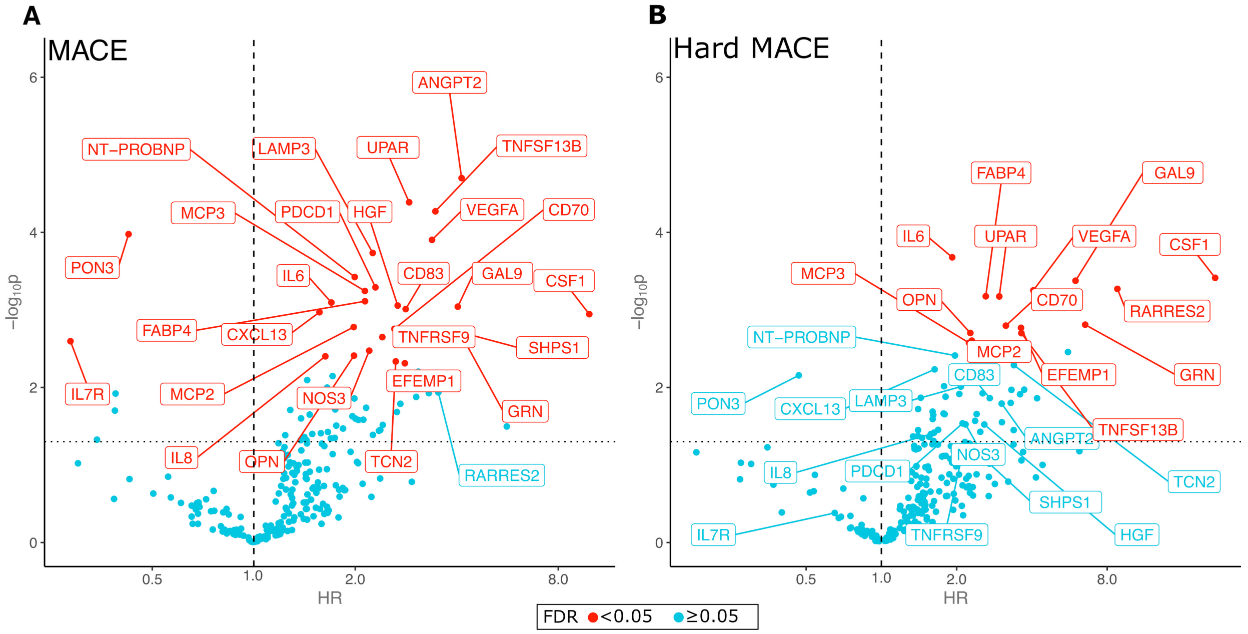

Panel A: Volcano plot of hazard ratios and p values for given proteins and MACE. Hazard ratios were estimated from Cox proportional hazard models using both potential proteomic measurements including statin randomization and baseline 10-year baseline ASCVD risk score. Hazard ratios are per protein doubling. Red proteins indicate associations at an FDR<0.05 level. X axis is on a $\log_2$ scale. Dotted line indicates a nominal p value of 0.05. Dashed line indicates a HR of 1.

Panel B: Volcano plot of hazard ratios and p values for given proteins and hard MACE. Hard MACE was defined as: cardiovascular death, myocardial infarction or stroke. Hazard ratios were estimated from Cox proportional hazard models using both potential proteomic measurements including statin randomization and baseline 10-year ASCVD risk score. Hazard ratios are per protein doubling. Red proteins indicate associations at an FDR<0.05 level. X axis is on a $\log_2$ scale. Dotted line indicates a nominal p value of 0.05. Dashed line indicates a HR of 1.

Abbreviations: ANGPT2, Angiopoietin-2; CD70, CD70 antigen; CD83, CD83 antigen; CSF1, Processed macrophage colony-stimulating factor 1; CXCL13, C-X-C motif chemokine 13; EFEMP1, EGF-containing fibulin-like extracellular matrix protein 1; FABP4, Fatty acid-binding protein, adipocyte; FDR, False discover rate; GAL9, Galectin-9; GRN, Paragranulin; HGF, Hepatocyte growth factor alpha chain; HR, Hazard ratio; IL6, Interleukin-6; IL7R, Interleukin-7 receptor subunit alpha; IL8, Interleukin-8; LAMP3, Lysosome-associated membrane glycoprotein 3; MACE, Major adverse cardiac event; MCP2, C-C motif chemokine 8; MCP3, C-C motif chemokine 7; NOS3, Nitric oxide synthase, endothelial; NT-PROBNP, NT-proBNP; OPN, Osteopontin; PDCD1, Programmed cell death protein 1; PON3, Serum paraoxonase/lactonase 3; RARRES2, Retinoic acid receptor responder protein 2; SHPS1, Tyrosine-protein phosphatase non-receptor type substrate 1; TCN2, Transcobalamin-2; TNFRSF9, Tumor necrosis factor receptor superfamily member 9; TNFSF13B, Tumor necrosis factor ligand superfamily member 13b, membrane form; UPAR, Urokinase plasminogen activator surface receptor; VEGFA, Vascular endothelial growth factor A

Supplemental figure 3. Forrest-plot of hazard ratios for proteins with a significant association with MACE or hard MACE in any of the statistical models.

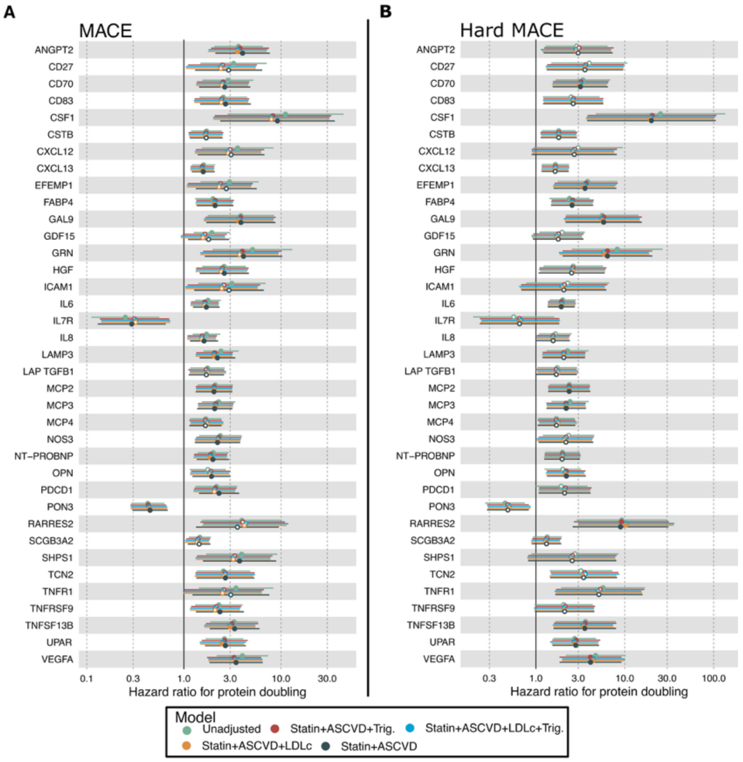

Panel A: Forrest plot of hazard ratios and confidence intervals for proteins' association with MACE. Hazard ratios were estimated from Cox proportional hazard models using both potential proteomic measurements adjusting for statin use, LDL-C and triglyceride levels as a time-dependent covariate and baseline 10-year baseline ASCVD risk score. Hazard ratios are per protein doubling. Any protein with FDR<0.05 association with the outcome is shown for all models. Full circles indicate FDR<0.05 level.

Panel B: Forrest plot of hazard ratios and confidence intervals for proteins' association with hard MACE. Hard MACE was defined as: cardiovascular death, myocardial infarction or stroke. Hazard ratios were estimated from Cox proportional hazard models using both potential proteomic measurements adjusting for statin use, LDL-C and triglyceride levels as a time-dependent covariate and baseline 10-year baseline ASCVD risk score. Hazard ratios are per protein doubling. Any protein with FDR<0.05 association with the outcome is shown for all models. Full circles indicate FDR<0.05 level.

Abbreviations: ANGPT2, Angiopoietin-2; CD27, CD27 antigen; CD70, CD70 antigen; CD83, CD83 antigen; CSF1, Processed macrophage colony-stimulating factor 1; CSTB, Cystatin-B; CXCL12, C-X-C motif chemokine 12; CXCL13, C-X-C motif chemokine 13; EFEMP1, EGF-containing fibulin-like extracellular matrix protein 1; FABP4, Fatty acid-binding protein, adipocyte; FDR, False discover rate; GAL9, Galectin-9; GDF15, Growth/differentiation factor 15; GRN, Paragranulin; IL6, Interleukin-6; IL7R, Interleukin-7 receptor subunit alpha; IL8, Interleukin-8; LAMP3, Lysosome-associated membrane glycoprotein 3; LAP TGFB1, Transforming growth factor beta-1 proprotein; LDLc, low-density lipoprotein cholesterol; MCP2, C-C motif chemokine 8; MCP3, C-C motif chemokine 7; MCP4, C-C motif chemokine 13, medium chain; NOS3, Nitric oxide synthase, endothelial; NT-PROBNP, NT-proBNP; OPN, Osteopontin; PDCD1, Programmed cell death protein 1; PON3, Serum paraoxonase/lactonase 3; RARRES2, Retinoic acid receptor responder protein 2; SCGB3A2, Secretoglobin family 3A member 2; SHPS1, Tyrosine-protein phosphatase non-receptor type substrate 1; TCN2, Transcobalamin-2; TNFRSF9, Tumor necrosis factor receptor superfamily member 9; TNFSF13B, Tumor necrosis factor ligand superfamily member 13b, membrane form; Trig: Triglyceride; UPAR, Urokinase plasminogen activator surface receptor; VEGFA, Vascular endothelial growth factor A

Supplemental figure 4. Correlation between baseline clinical characteristics and proteins showing a significant association with MACE and ANGPTL3.

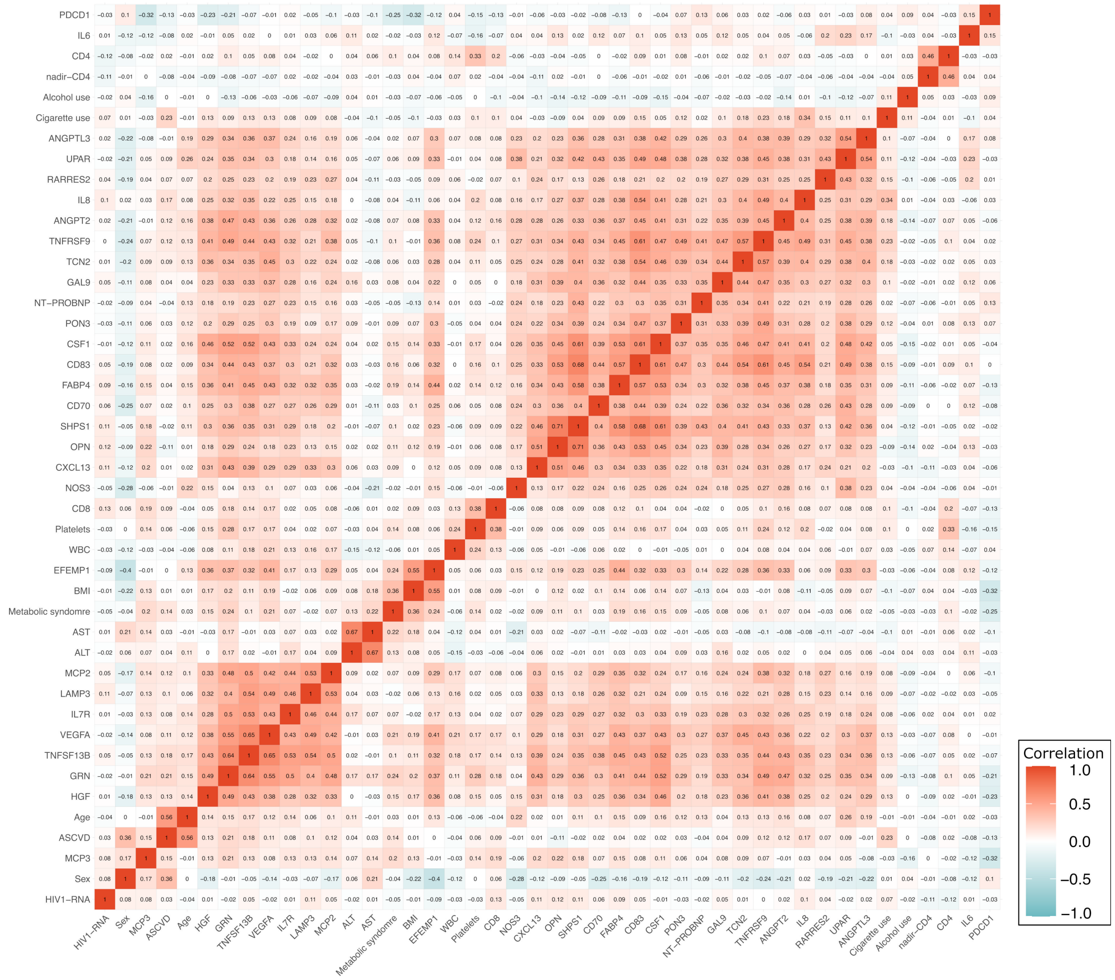

Baseline values using blood samples prior to randomization were used. Correlation values were estimated using Spearman's or point-biserial correlation, as appropriate. Cigarette smoking was defined as current or former smoker. Alcohol use was defined as usually, often or sometimes based on self-questionaries.

Abbreviations: ANGPT2, Angiopoietin-2; ANGPTL3, Angiopoietin-related protein 3; ALT, Alanine transaminase; AST, Aspartate transferase; ASCVD, 10-year atherosclerotic cardiovascular disease risk score; BMI: Body mass index; CD4, CD4 antigen; CD70, CD70 antigen; CD8, CD8 antigen; CD83, CD83 antigen; CSF1, Processed macrophage colony-stimulating factor 1; CXCL13, C-X-C motif chemokine 13; EFEMP1, EGF-containing fibulin-like extracellular matrix protein 1; FABP4, Fatty acid-binding protein, adipocyte; FDR, False discover rate; GAL9, Galectin-9; GRN, Paragranulin; HDL: high-density lipoprotein; HGF, Hepatocyte growth factor alpha chain; hsCRP, high sensitivity C-reactive protein; IL6, Interleukin-6; IL7R, Interleukin-7 receptor subunit alpha; IL8, Interleukin-8; LAMP3, Lysosome-associated membrane glycoprotein 3; LDL-C, low-density lipoprotein cholesterol; Lp-PLA 2, lipoprotein-associated phospholipase A2; MCP2, C-C motif chemokine 8; MCP3, C-C motif chemokine 7; NOS3, Nitric oxide synthase, endothelial; NT-PROBNP, NT-proBNP; OPN, Osteopontin; PDCD1, oxLDL, oxidized low-density lipoprotein; Programmed cell death protein 1; PON3, Serum paraoxonase/lactonase 3; RARRES2, Retinoic acid receptor responder protein 2; SHPS1, Tyrosine-protein phosphatase non-receptor type substrate 1; TCN2, Transcobalamin-2; TNF, Tumor necrosis factor; TNFRSF9, Tumor necrosis factor receptor superfamily member 9; TNFSF13B, Tumor necrosis factor ligand superfamily member 13b, membrane form; UPAR, Urokinase plasminogen activator surface receptor; VEGFA, Vascular endothelial growth factor A; WBC, white blood cell

Supplemental figure 5. Correlation between baseline lipid, biomarkers, and proteins showing a significant association with MACE and ANGPTL3.

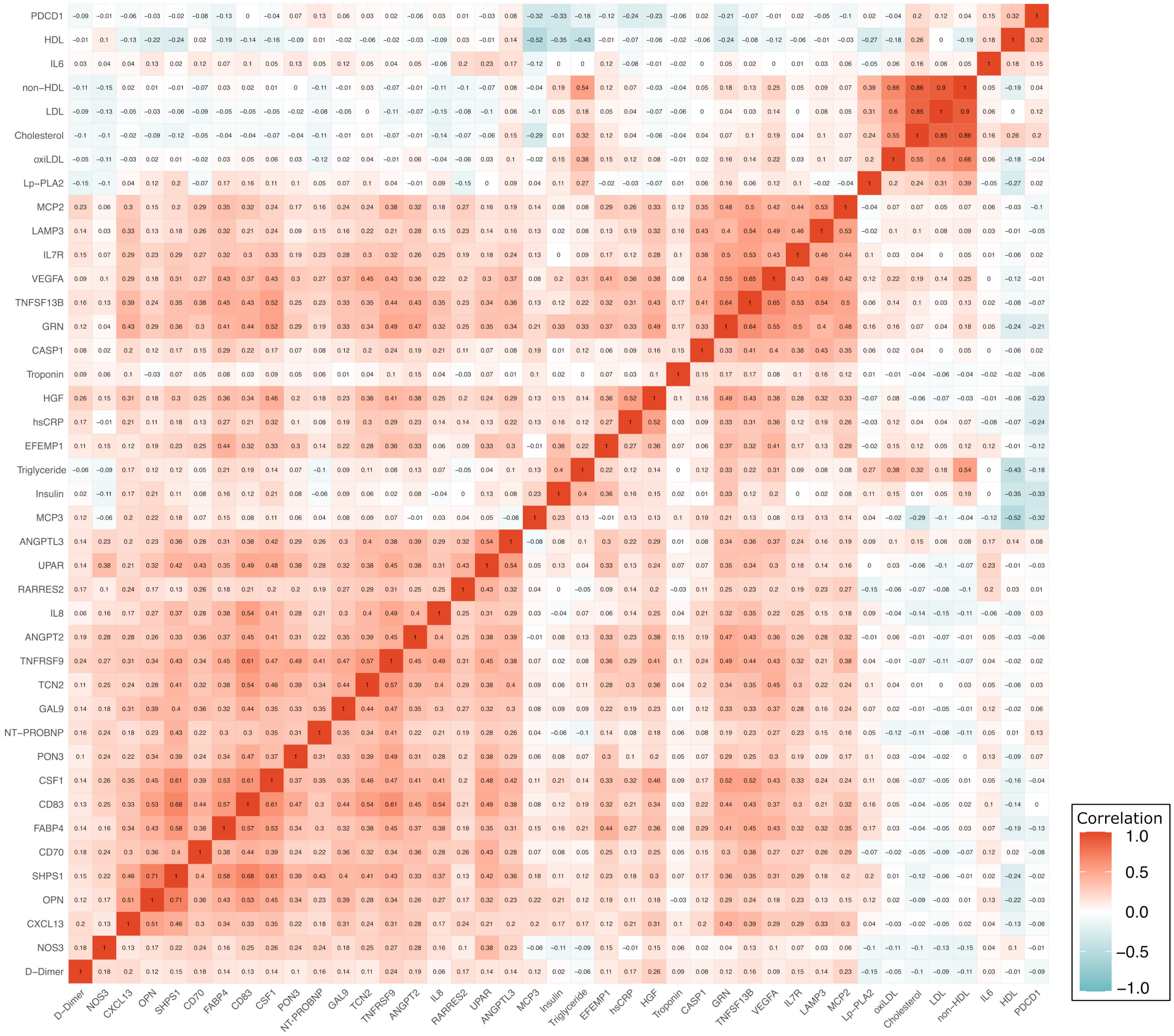

Baseline values using blood samples prior to randomization were used. Correlation values were estimated using Spearman's or point-biserial correlation, as appropriate.

Abbreviations: ANGPT2, Angiopoietin-2; ANGPTL3, Angiopoietin-related protein 3; ALT, Alanine transaminase; AST, Aspartate transferase; ASCVD, 10-year atherosclerotic cardiovascular disease risk score; BMI: Body mass index; CD4, CD4 antigen; CD70, CD70 antigen; CD8, CD8 antigen; CD83, CD83 antigen; CSF-1, Processed macrophage colony-stimulating factor 1; CXCL13, C-X-C motif chemokine 13; EFEMP1, EGF-containing fibulin-like extracellular matrix protein 1; FABP4, Fatty acid-binding protein, adipocyte; FDR, False discover rate; GAL9, Galectin-9; GRN, Paragranulin; HDL: high-density lipoprotein; HGF, Hepatocyte growth factor alpha chain; hsCRP, high sensitivity C-reactive protein; IL6, Interleukin-6; IL7R, Interleukin-7 receptor subunit alpha; IL8, Interleukin-8; LAMP3, Lysosome-associated membrane glycoprotein 3; LDL-C, low-density lipoprotein cholesterol; Lp-PLA 2, lipoprotein-associated phospholipase A2; MCP2, C-C motif chemokine 8; MCP3, C-C motif chemokine 7; NOS3, Nitric oxide synthase, endothelial; NT-PROBNP, NT-proBNP; OPN, Osteopontin; PDCD1, oxLDL, oxidized low-density lipoprotein; Programmed cell death protein 1; PON3, Serum paraoxonase/lactonase 3; RARRES2, Retinoic acid receptor responder protein 2; SHPS1, Tyrosine-protein phosphatase non-receptor type substrate 1; TCN2, Transcobalamin-2; TNF, Tumor necrosis factor; TNFRSF9, Tumor necrosis factor receptor superfamily member 9; TNFSF13B, Tumor necrosis factor ligand superfamily member 13b, membrane form; UPAR, Urokinase plasminogen activator surface receptor; VEGFA, Vascular endothelial growth factor A; WBC, white blood cell
